# Co-culturing hiPSC-cardiomyocytes and cardiac fibroblasts enhances engineered heart tissue structure and function

**DOI:** 10.1093/stcltm/szag041

**Published:** 2026-06-27

**Authors:** Yinhan Luo, Jeremy Parker, Armando Alcázar Magaña, Ardin Sacayanan, Kate Huang, Ian Fernandes, Gordon M Keller, Peter H Backx, Leonard J Foster, Zachary Laksman

**Affiliations:** Centre for Heart Lung Innovation, University of British Columbia, Vancouver V6Z 1Y6, Canada; Department of Experimental Medicine, University of British Columbia, Vancouver V6T 1Z4, Canada; Centre for Heart Lung Innovation, University of British Columbia, Vancouver V6Z 1Y6, Canada; School of Biomedical Engineering, University of British Columbia, Vancouver V6T 1Z4, Canada; Department of Biochemistry & Molecular Biology, Michael Smith Laboratories, University of British Columbia, Vancouver V6T 1Z4, Canada; Centre for Heart Lung Innovation, University of British Columbia, Vancouver V6Z 1Y6, Canada; School of Biomedical Engineering, University of British Columbia, Vancouver V6T 1Z4, Canada; Centre for Heart Lung Innovation, University of British Columbia, Vancouver V6Z 1Y6, Canada; Department of Experimental Medicine, University of British Columbia, Vancouver V6T 1Z4, Canada; Division of Cardiology, Department of Medicine, University of British Columbia, Vancouver V6T 1Z4, Canada; McEwen Stem Cell Institute, University Health Network, Toronto M5G 1L7, Canada; University of Toronto, Toronto M5S 3H2, Canada; McEwen Stem Cell Institute, University Health Network, Toronto M5G 1L7, Canada; University of Toronto, Toronto M5S 3H2, Canada; Department of Biology, York University, Toronto M3J 1P3, Canada; Life Sciences Institute, University of British Columbia, Vancouver V6T 1Z4, Canada; Centre for Heart Lung Innovation, University of British Columbia, Vancouver V6Z 1Y6, Canada; Department of Experimental Medicine, University of British Columbia, Vancouver V6T 1Z4, Canada; School of Biomedical Engineering, University of British Columbia, Vancouver V6T 1Z4, Canada; Division of Cardiology, Department of Medicine, University of British Columbia, Vancouver V6T 1Z4, Canada

**Keywords:** stem cell, engineered heart tissue, 3-dimensional cardiac model, cardiac electrophysiology, cardiac metabolism

## Abstract

**Background:**

Engineered heart tissues (EHTs) are widely used for cardiac disease modeling and drug screening, but their lack of multicellularity limits translational relevance. Thus, it is essential to incorporate other cardiac cells to improve the reliability and accuracy of the model.

**Objectives:**

To develop a co-culture EHT model from human induced pluripotent stem cell (hiPSC)-derived cardiomyocytes (CMs) and cardiac fibroblasts (CFs) and assess its structural, contractile, electrophysiological and metabolic properties.

**Methods:**

hiPSCs were differentiated into CMs and CFs and combined at a ratio of 3:1 to generate co-culture EHTs. Structural, functional and metabolic features of CM-only and co-culture EHTs were evaluated and compared using immunofluorescence, force analysis, optical mapping and metabolomics.

**Results:**

Co-culture EHTs were more compact, generated higher force when stimulated, and displayed improved sarcomere organization compared to CM-only EHTs. They showed reduced hypoxia under high frequency pacing and a more mature, stress resistant metabolic profile, while maintaining stable electrophysiology and reduced arrhythmogenicity.

**Conclusions:**

Incorporating hiPSC-CFs into EHTs enhanced structural and functional properties, improved stress resistance, and reduced variability, making our co-culture EHTs a more physiological and predictive platform for cardiac disease modeling and drug screening.

## Graphical Abstract

**Graphical Abstract:**
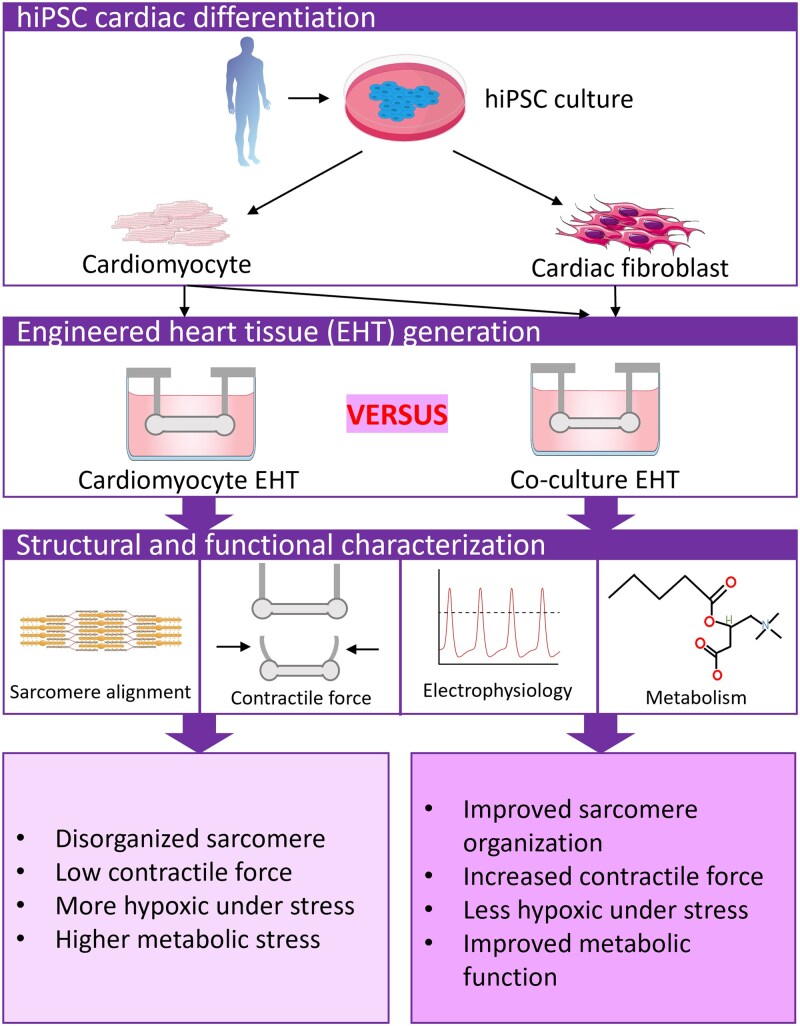

Significance Co-culture engineered heart tissues (EHTs) more closely recapitulate key features of the adult human heart, including cellular composition, tissue organization, and metabolic behaviour, thereby narrowing the gap between *in vitro* models and native cardiac physiology. These tissues exhibit enhanced contractile force, reduced hypoxia, and improved resilience under pacing stress, making them more robust and reproducible platforms for modeling cardiac diseases involving multicellular interactions and advancing translational cardiac research.

## Introduction

Induced pluripotent stem cell-derived cardiomyocytes (hiPSC-CMs) are widely used for patient-specific disease modeling and drug screening.^1–3^ Advances in tissue engineering have enabled the development of 3-dimensional cardiac models such as fibrin-based engineered heart tissue (EHT), which promote maturation of the cells and enable functional measurements, including contractile force with and without electrical stimulation.^4^^,^^5^ EHTs reliably capture disease phenotypes and pharmacological responses.^5^^,^^6^ However, their translational relevance is limited by insufficient multicellularity, as cardiomyocytes (CMs) only represent approximately 30% of the cell population of the human heart^7^ despite occupying most of the myocardial volume.^7–9^ To address this limitation, cardiac fibroblasts (CFs)^10–12^, endothelial cells,^13^^,^^14^ or both^15^ into the 3-dimensional model to recapitulate the native myocardial environment.

CFs are the primary source of extracellular matrix (ECM) and are essential for maintaining myocardial structure.^16^ They promote CM maturation and alignment via ECM production, paracrine signaling, and cell-cell interactions.^16^^,^^17^ CFs also modulate electrical activity via gap-junction coupling with CMs.^17^^,^^18^ Critically, CFs influence CM metabolism by regulating the energy source utilization^19^^,^^20^ and responses to oxidative stress.^21^^,^^22^ Despite their importance in cardiac physiology and disease modeling, the contribution of CFs has not been thoroughly examined in hiPSC-based cardiac models, particularly in EHT, which are increasingly used for disease modeling and drug screening.

In this study, we established a co-culture EHT model by combining hiPSC-CMs with hiPSC-CFs at a 3:1 ratio. Using hiPSC lines from both male and female donors, we compared co-culture EHTs and CM-only EHTs with respect to contractile force, structural organization, electrophysiology and metabolism. Co-culture EHTs generated significantly higher contractile force, exhibited improved sarcomere alignment, and displayed reduced variability between batches and tissues. Under stress conditions, they were less hypoxic and demonstrated and enhanced fatty acid oxidation as compared with CM-only EHTs. Together, these findings showed that co-culture EHTs more faithfully capture the multicellular environment of the human heart and provide a more physiological and reliable *in vitro* platform for cardiac disease modeling and drug testing.

## Methods

All experimental procedures and methodological details are described in the Supplementary Materials.

## Results

### hiPSC-CMs and hiPSC-CFs were differentiated and expressed cell-specific markers

**Figure 1.**
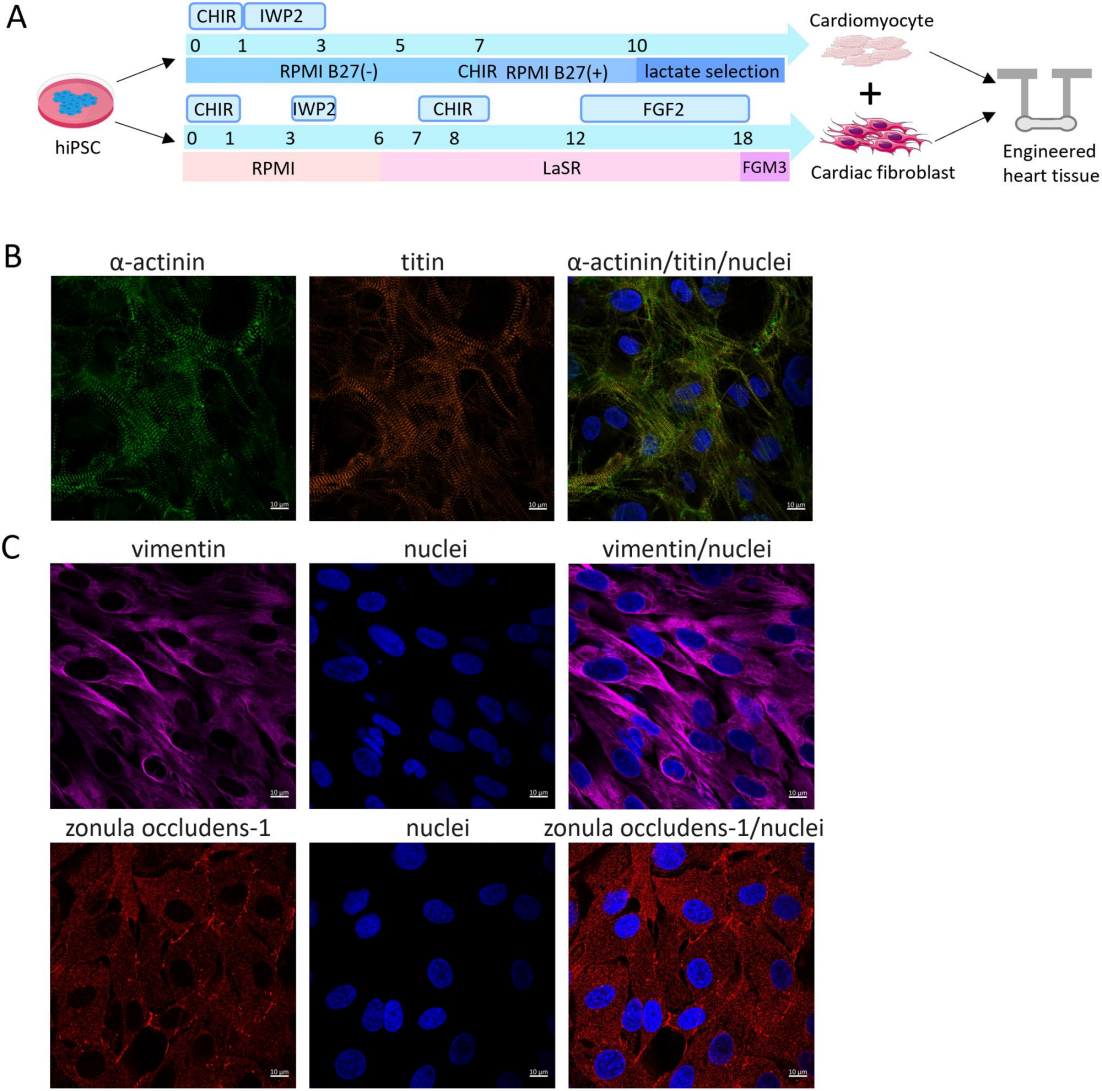
Differentiation of hiPSC-CMs and hiPSC-CFs and quality control assays. (A) Schematic diagram of hiPSC-CM and hiPSC-CF differentiation protocols. The cell culture media, small molecules, and differentiation steps are indicated. Icons used in the diagram were taken from Bioicons. (B) Immunofluorescence images showing the expression of sarcomere proteins α-actinin (green) and titin (orange) in hiPSC-CMs from line 1. Nuclei were counterstained with Hoechst (blue). (C) Immunofluorescence images of hiPSC-CFs stained for fibroblast markers vimentin (magenta) and zonula occludens-1 (red). Nuclei were counterstained with Hoechst (blue). Abbreviation: hiPSC, human induced pluripotent stem cell.

We differentiated CMs and CFs from a male- and a female-derived hiPSC line using small molecule-based protocols (Figure 1A). To ensure CM purity, we assessed cardiac troponin T (cTnT) expression (Supplementary Figure 1A) and only used CM populations with >70% cTnT to generate EHTs. hiPSC-CMs expressed sarcomere proteins α-actinin and titin (Figure 1B) while hiPSC-CFs expressed the structural protein vimentin and tight junction protein Zonula occludens-1 (Figure 1C). Gene expression analysis further confirmed that the CMs expressed pan-cardiac genes *TNNT2* and *TBX5* and ventricular genes *MYL2* and *IRX4* (Supplementary Figure 1B) while the CFs expressed intermediate filament gene *VIM* and extracellular matrix genes *POSTN*, *COL1A2* and *FN1* (Supplementary Figure 1C; primers are listed in Supplementary Table 1).

### Co-culture EHTs were more compact than CM-only EHTs

**Figure 2.**
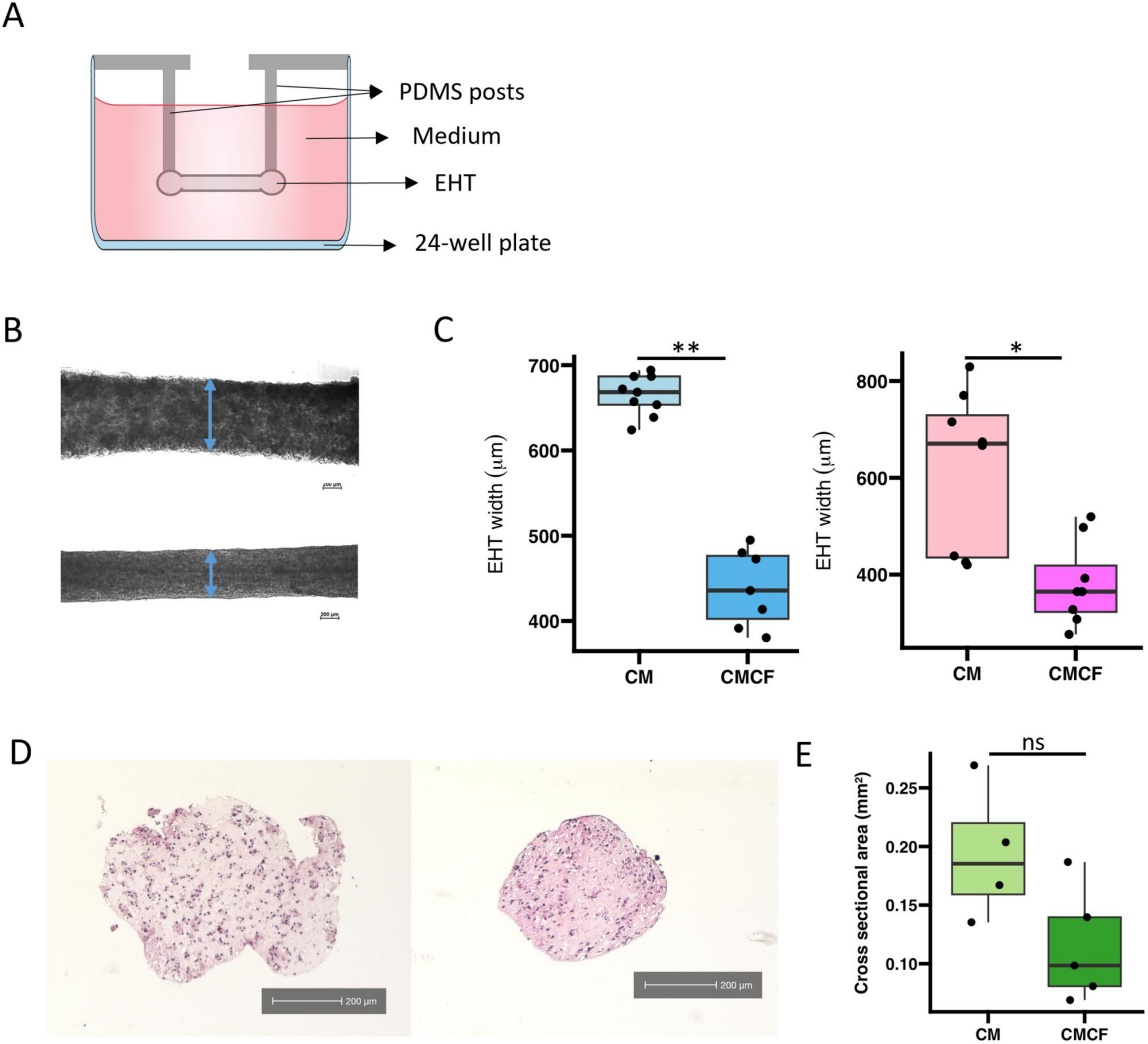
CM-only versus co-culture EHT morphology and cross-sectional area measurements. (A) Schematic diagram of an EHT cultured in a 24-well plate. (B) Microscopic images of a CM EHT (left) and a co-culture EHT (right). Width measurements were taken in the middle of the tissue and marked by the blue arrows. (C) Average widths of CM-only EHTs versus co-culture EHTs from line 1 and line 2. Data are presented as median ± IQR; *t*-test and Wilcoxon test. (D) Microscopic images of the cross section of a CM-only (left) EHT and a co-culture (right) EHT stained with hematoxylin and eosin. (E) Quantification of the cross-sectional area of CM-only vs co-culture EHTs. Data are presented as median ± IQR; *t*-test. Statistical significance: *P < .05; **P < .01. Abbreviations: PDMS, Polydimethylsiloxane; EHT, Engineered heart tissue; CM, cardiomyocyte-only; CMCF, co-culture.

CM-only EHTs were generated with hiPSC-CMs, whereas co-culture EHTs were generated by combining hiPSC-CMs and hiPSC-CFs at a ratio of 3:1 (Figure 2A). Each EHT contained 800 000 cells in total and spontaneous contraction was observed within a week post casting. hiPSC-CFs were evenly distributed throughout the tissue as demonstrated using GFP-tagged CFs in the EHTs (Supplementary Figure 2A). To assess the difference in morphology, we measured the width at the center of CM-only versus co-culture EHTs (Figure 2B) and co-culture EHTs appeared to be thinner and more compact. In both line 1 and line 2, CM-only EHTs were significantly wider than co-culture EHTs (Figure 2C; mean 665 μm versus 438 μm, *P *= .001; mean 618 μm versus 381 μm, *P *= .005). We further evaluated structural differences by sectioning and performing hematoxylin and eosin staining of the EHTs (Figure 2D) to assess the cross-sectional area and visualize the cell distribution in the tissue. Although the difference did not reach statistical significance, co-culture EHTs showed a smaller cross-sectional area than that of CM-only EHTs (Figure 2E; mean 0.194 mm^2^ versus 0.115 mm^2^, *P *= .07). The average cross-sectional area of CM-only and co-culture EHTs were calculated and subsequently used to normalize contractile force measurements.

### Co-culture EHTs exhibited higher force than CM-only EHTs when electrically stimulated

**Figure 3.**
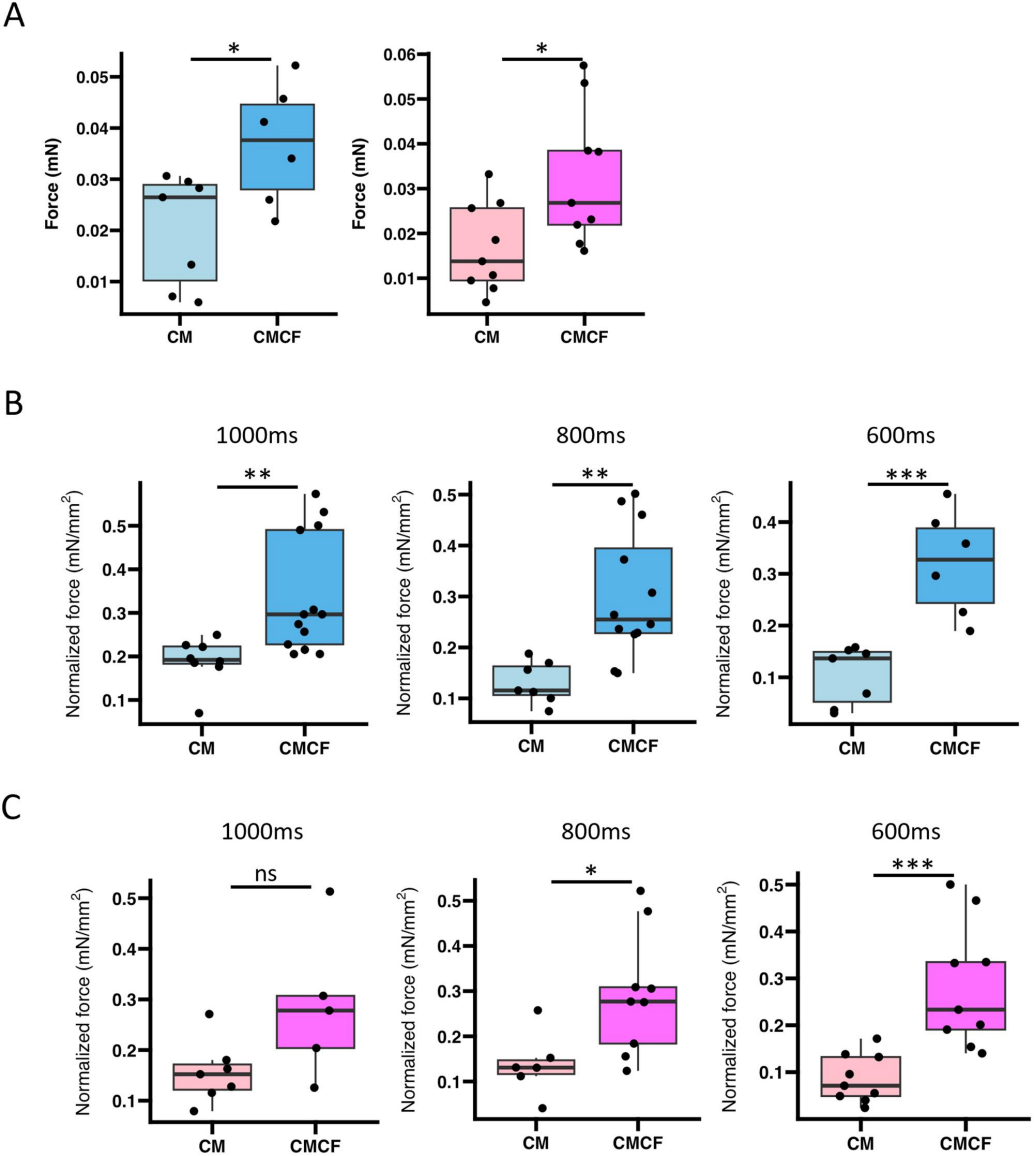
EHT unnormalized and normalized force measurements under electrical stimulation. (A) Contractile force generated by CM-only EHTs versus co-culture EHTs from line 1 and line 2 paced at 600 ms. Blue: line 1, pink: line 2. (B) Contractile force of EHTs normalized to the average cross-sectional areas from line 1 and (C) line 2 paced at 1000 ms, 800 ms and 600 ms. Data are presented as median ± IQR; Welch test; Statistical significance: *P < .05; **P < .01; ***P < .001. Abbreviations: CM, cardiomyocyte-only; CMCF, co-culture.

Contractility is a fundamental indicator of cardiac function. To determine whether differences in tissue compactness would lead to altered contractile force, we electrically paced the EHTs at increasing frequencies using a custom-built stimulator (Supplementary Figure 2B) and measured the generated force. In both hiPSC lines, co-culture EHTs produced significantly higher force than CM-only EHTs when stimulated at 600 ms (Figure 3A; mean 0.020 mN versus 0.037 mN, *P *= .023; mean 0.017 mN versus 0.033 mN, *P *= .018), whereas no differences were observed at lower pacing frequencies of 1000 ms and 800 ms (*P* > 0.05, data not shown in the figure). When force was normalized to cross-sectional area, co-culture EHTs demonstrated significantly greater force than CM-only EHTs at 1000 ms, 800 ms and 600 ms in line 1 (Figure 3B, mean 0.189 mN/mm^2^ versus 0.337 mN/mm^2^, *P *= .001; mean 0.131 mN/mm^2^ versus 0.303 mN/mm^2^, *P *= .003; mean 0.104 mN/mm^2^ versus 0.320 mN/mm^2^, *P *= 5.262×10^−4^) and 800 ms and 600 ms and in line 2 (Figure 3C, mean 0.137 mN/mm^2^ versus 0.292 mN/mm^2^, *P *= .024; mean 0.086 versus 0.284, *P *= 7.231262×10^−4^). These findings indicate that the inclusion of CFs enhanced contractile performance of the EHTs at physiological beat rates.

### Co-culture EHTs exhibited improved sarcomere alignment and are less hypoxic than CM-only EHTs under stress

**Figure 4.**
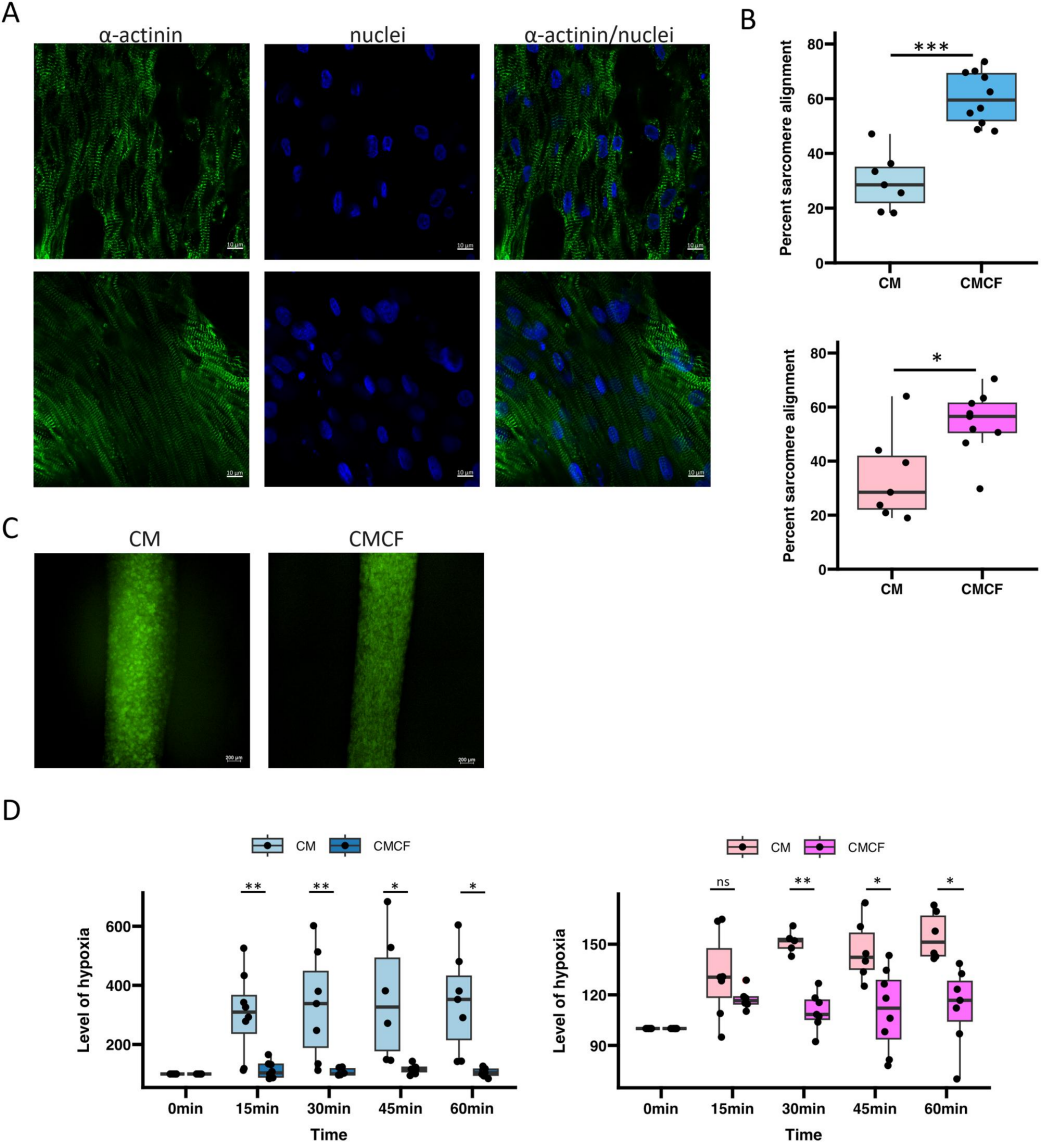
Sarcomere alignment and hypoxic levels of CM-only versus co-culture EHTs. (A) CM-only EHT (top) and co-culture EHT (bottom) stained with anti-α-actinin (green) and Hoechst (blue) and imaged at 63×. (B) Percent sarcomere alignment of CM-only and co-culture EHTs within 10 degrees. Line 1(top) line 2 (bottom). Data are presented as median ± IQR; *t*-test. (C) Fluorescent images of a CM-only EHT and a co-culture EHT stained with hypoxia stain and paced at 400 ms for 60 min. (D) Level of hypoxia of CM-only versus co-culture EHTs at various timepoints under rapid pacing. Line 1(top) line 2 (bottom). Data are presented as median ± IQR; *t*-test and Wilcoxon test. Statistical significance: *P < .05; **P < .01; ***P < .001. Abbreviations: CM, cardiomyocyte-only; CMCF, co-culture.

The sarcomere is the fundamental unit of contraction in CMs and is responsible for force generation. We hypothesized that the difference in force generation between the CM-only and co-culture EHTs could be attributed to variations in their sarcomere structure. To examine this, we first visualized sarcomere organization by staining the EHTs with an anti-α-actinin antibody (Figure 4A). The alignment was then quantified by analyzing the directionality of the sarcomere. Co-culture EHTs displayed significantly improved alignment, with higher alignment scores compared to CM-only EHTs (Figure 4B; mean 29.68% versus 60.27%, *P *= 1.451e−05; mean 34.21% versus 54.245%, *P *= .012; Supplementary Figures 2C and D). In addition, we performed qPCR to test whether co-culture EHTs express the more mature isoforms of sarcomere genes. Both types of EHTs expressed comparable levels of *MYL7* and *TNNC1*, while co-culture EHTs showed visually higher, though not significantly different expression of *MYL2* and *TNNI3* (Supplementary Figure 3A)**—**markers associated with CM maturation.

Because the thinner, more compact co-culture EHTs exhibited higher force than the thicker CM-only EHTs, we next asked whether improved cell compaction and alignment facilitates oxygen diffusion. To test this, we stained the EHTs with hypoxia-sensitive dye before stimulating them at 400 ms (150 beats per minute) for 1 h (Figure 4C). In CM-only EHTs, hypoxia progressively increased over time, whereas levels remained relatively stable in co-culture tissues. In line 1, CM-only EHTs were significantly more hypoxic than co-culture ones at every time point after stimulation (Figure 4D, *P *= .0062, .0027, .049, .026). Similarly, CM-only EHTs were more hypoxic than their co-culture counterparts at 30 min, 45 min and 60 min timepoints in line 2 (Figure 4D, *P *= .0037, .012, .042). Notably, in line 1, the variance of hypoxia levels was significantly greater in the CM-only EHTs compared to co-culture EHTs at 15 min, 45 min and 60 min timepoints (Supplementary Table 2; Levene’s test; *P *= .024, .025 and .05).

### Electrophysiological characterization of CM-only versus co-culture EHTs

**Figure 5.**
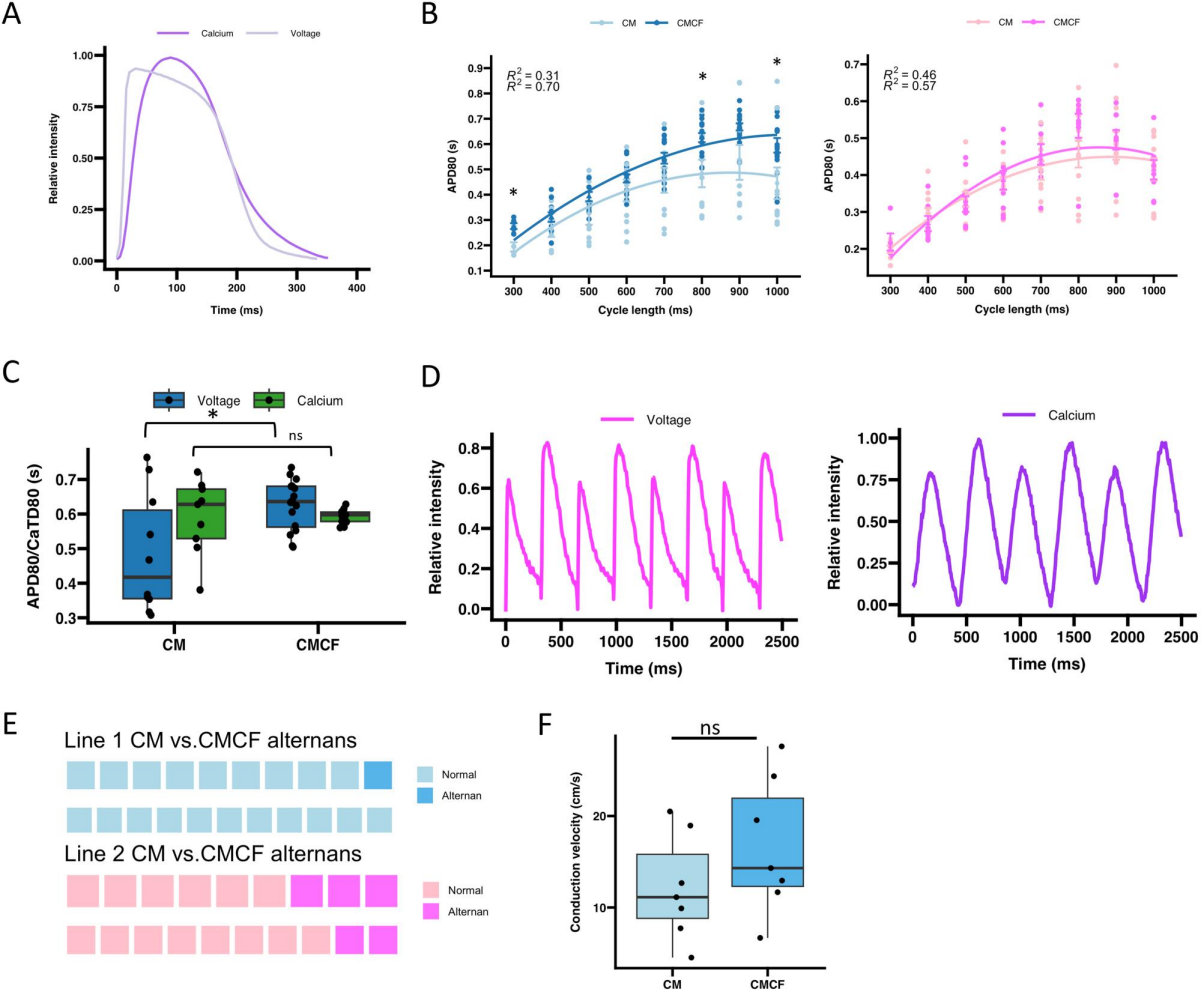
Electrophysiological properties of EHTs under electrical stimulation. (A) Representative voltage and calcium transients of a co-culture EHT. (B) APD80 restitution curves of CM-only versus co-culture EHTs. The curves were fitted to second degree polynomial equations. The cycle lengths that show a significant difference are indicated by the asterisks. Line 1(left), line 2 (right). *T*-test and Wilcoxon test. (C) APD80 and CaTD80 of line 1 EHTs paced at 800 ms. (D) Representative traces of voltage and calcium alternans observed in CM-only EHTs from line 2. (E) Waffle plot showing the number of EHTs that exhibited alternans. Top row: CM-only EHTs; bottom row: co-culture EHTs. (F) Conduction velocity of CM-only and co-culture EHTs paced at 700 ms. Data are presented as median ± IQR; t-test. Statistical significance: *P < .05. Abbreviations: CM, cardiomyocyte-only; CMCF, co-culture.

Next, we performed optical mapping to characterize the electrophysiological properties of the EHTs. The EHTs exhibited normal ventricular voltage and calcium transients under electrical stimulation (Figure 5A). We paced the tissues at progressively increasing frequencies and performed optical mapping to measure the action potential durations (APDs) and calcium transient durations (CaTDs). The restitution curve profiles were fitted to second degree polynomial equations (Line 1: CM *y* = 0.404 + 0.744*x−*0.362*x*^2^, CMCF *y* = 0.517 + 1.21*x−*0.34*x*^2^; line 2: CM *y* = 0.379 + 0.652*x−*0.288*x*^2^, CMCF *y* = 0.383 + 0.6*x−*0.329*x*^2^) and were comparable in shape between CM-only and co-culture EHTs (Figure 5B). Notably, in line 1, co-culture EHTs displayed significantly longer APD at 80% repolarization (APD80) values than CM-only EHTs at cycle lengths of 1000 ms, 800 ms and 300 ms (Figure 5C; Wilcoxon test and *t*-test; *P *= .027, .032, and .015). Also, co-culture EHTs in line 1 showed longer APD at 50% repolarization (APD50) values at 300 ms and CM-only EHTs showed longer APD50 values at 400 ms and 1000 ms (Supplementary Figure 3B; Mann-Whitney *U* test and *t*-test; *P *= .007, .008, and .028). In contrast, CaTDs were not significantly different between the two types of EHTs (Supplementary Figure 3C). Variance analysis further revealed that APD80 values were more consistent in co-culture EHTs compared to CM-only EHTs across both lines (Supplementary Table 3). In line 1, significant reductions in variability were observed at 900 ms, 800 ms and 600 ms (Levene’s test; *P *= .0006, .011, and .010), and in line 2 at 900 ms, 800 ms, and 400 ms (Levene’s test; *P *= .028, .022, and .035).

We also assessed voltage and calcium alternans, proarrhythmic features that can emerge under stress conditions such as rapid pacing (Figure 5D). Although the incidence of alternans appeared lower in co-culture EHTs for both lines, the differences did not reach statistical significance (Figure 5E). Finally, conduction velocity tended to be higher in co-culture EHTs compared to CM-only EHTs when paced at 700 ms, but it was not statistically significant (Figure 5F).

### Metabolomic profiling of CM-only EHTs versus co-culture EHTs

As the cardiac tissue matures, it transitions from primarily utilizing glucose to relying on fatty acid as the main energy source, with a concomitant increase in oxidative phosphorylation.^23^ We hypothesized that the co-culture EHTs were more mature, resembling adult cardiac tissues, and therefore better equipped to withstand stress. To assess metabolic differences between CM-only and co-culture EHTs, we performed untargeted metabolomics on both tissue types. Differentially expressed metabolites were summarized in volcano plots (Supplementary Tables 4 and 5).

**Figure 6.**
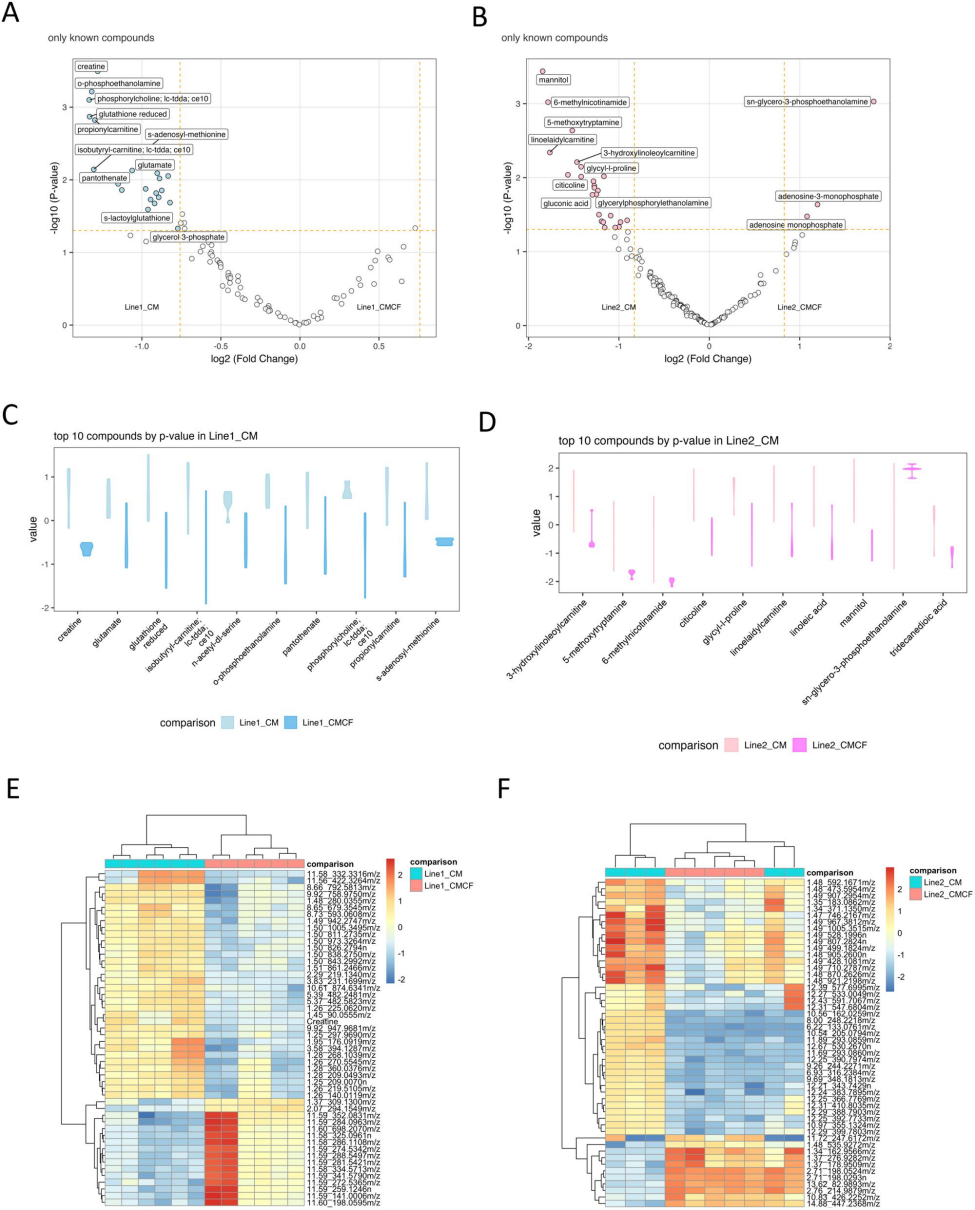
Metabolic profiles of CM-only EHTs vs. co-culture EHTs. (A) Volcano plot showing differentially expressed metabolites (known compounds only) between CM-only and co-culture EHTs from line 1 and (B) line 2. Statistically significant differentially expressed metabolites are in blue (line 1) and pink (line 2) (C) Violin plots showing the top 10 differentially expressed compounds in CM-only and co-culture EHTs from line 1 and (D) line 2. (E) Heatmaps of metabolites identified in CM-only and co-culture EHTs from line 1 and (F) line 2. Abbreviations: CM, cardiomyocyte-only; CMCF, co-culture.

In line 1, 27 metabolites were significantly upregulated in CM-only EHTs compared to co-culture EHTs (Figure 6A). Meanwhile in line 2, 3 out of the 27 metabolites were upregulated in CMCF EHTs (Figure 6B). A notable finding was the enrichment of multiple forms of acylcarnitine, particularly long chain acylcarnitine (LCAC), in CM-only EHTs. The top 10 differentially expressed metabolites in line 1 included creatine, glutathione and its precursors, several species of acylcarnitine and phospholipids (Figure 6C). In line 2, metabolites such as 5-methoxytrptamine, citicholine and long chain fatty acids were elevated in CM-only EHTs, with acylcarnitines being the most prominent one, and AMP was upregulated in co-culture EHTs (Figure 6D). Heatmap analysis further revealed distinct metabolic profiles between the two tissue types, with samples clustering according to their group identity (Figures 6E and F).

## Discussion

In this study, we co-cultured hiPSC-derived CMs and CFs in an EHT model to generate a preclinical cardiac model with physiologically relevant structure, function and energy metabolism. CFs were included due to the their essential roles in ECM deposition, tissue organization and dynamic biochemical, electrical and biomechanical interactions with CMs.^24^ A CM to CF ratio of 3:1 was selected based on single-cell analyses of the human ventricle, which report that CMs and CFs comprise ∼49.2% and ∼15.5% of the cell population, respectively.^18^ We chose the fibrin-based EHT platform because of its significant practical advantages. This system requires minimal instrumentation, relies entirely on commercially available components, and can be readily implemented in any standard laboratory equipped with basic tissue culture facilities. These features make it highly accessible, cost-effective, and reproducible across laboratories. In contrast, bioprinting and BioWire-based platforms depend on specialized equipment and customized hardware and software, which may limit their accessibility and scalability.

### Tissue morphology and compaction

Morphological analysis revealed striking differences between CM-only and co-culture EHTs. Despite having the same number of cells, co-culture tissues were significantly thinner with smaller cross-sectional areas, reflecting greater tissue compaction. This observation is consistent with an earlier study using embryonic stem cell-derived CFs in the bio-wire platform, where the addition of 25% CFs facilitated compaction and reduced tissue width to 0.3-0.4 mm.^25^ Similarly, bio-printed co-culture EHTs incorporating primary human CFs demonstrated improved cellular alignment and tissue compaction compared with the CM-only controls.^26^ In addition, CM-CF co-cultures at multiple ratios showed anisotropic organization under uniaxial cyclic strain, whereas CMs alone remained disorganized.^27^ Beyond morphological remodeling, CFs actively produce, remodel and degrade ECM thereby adjusting the stiffness of the myocardium and maintaining homeostasis in the tissue.^9^ The deposition of collagen by the CFs contributed to tissue compaction, a sign of structural maturation, in the EHTs.^28^ Moreover, paracrine signaling between CMs and CFs also likely contributes to improved viability and structural integrity of the co-culture EHTs. CFs secrete many interleukins (ILs) that modulate CM behaviour.^17^ For example, IL-33, which is predominantly synthesized by CFs, has been shown to attenuate pressure overload-induced hypertrophy and fibrosis.^29^^,^^30^ In addition, IL-6 secretion is increased in CM-CF co-culture systems, where it has been reported to promote ECM turnover and protect cells from apoptosis.^31^ These findings underscore that hiPSC-CFs recapitulate the structural effects observed with embryonic and primary CFs.

### EHT force generation under pacing

We hypothesized that enhanced compaction improves cell-cell communication via gap junctions and facilitates mechanical signal transmission, resulting in greater force generation in co-culture EHTs. Unlike the adult heart, which relies on electrical activation, the embryonic heart responds to mechanical signals,^32^ a process in which mechanosensitive CFs play a central role. CF-mediated transmission of mechanical cues and upregulation of ECM components such as collagen I, together with increased expression increase expression of contractile proteins in the CMs, promote ECM stiffening and improved tissue performance.^33^^,^^34^ Indeed, co-culture EHTs generated higher contractile force across pacing frequencies than CM-only EHTs, and expressed higher levels of maturation markers *MYL2* and *TNNI3*. Moreover, while CM-only EHTs displayed a negative force-frequency relationship, consistent with the spheroid model,^11^ co-culture EHTs maintained stable force output at higher pacing frequencies. This stability indicates greater robustness and resistance to stress, a hallmark of adult myocardium.^35^ The improved contractile machinery in co-culture tissues may also be supported by membrane nanotube-mediated Ca^2+^ exchange and mitochondria transfer between CMs and CFs.^18^^,^^36^

The increased contractile force observed in our co-culture EHTs represents a key advantage for disease modeling and drug screening, providing a more physiological baseline for detecting disease-associated impairments and evaluating inotropic drug responses. As previously reported,^5^^,^^37^^,^^38^ changes in contractility in EHTs can be subtle and difficult to resolve. The higher baseline force of co-culture EHTs improves sensitivity to both positive and negative inotropic effects, enhancing their translational potential.

### Improved sarcomere alignment in co-culture EHTs

The sarcomere is the fundamental unit of contraction in the cardiac tissues^39^ and improved sarcomere organization is a hallmark of CM maturation.^40^^,^^41^ After observing the increased contractility of co-culture EHTs, we asked whether this was due to improved sarcomere alignment. CFs secrete and remodel ECM components, particularly collagen, which increases the stiffness of the environment.^16^^,^^27^ CMs sense the mechanical and biochemical cues through mechanotransducive pathways, leading to enhanced sarcomere organization-a hallmark of structural maturation.^34^^,^^42^ Previous studies have also demonstrated that CFs promote CM maturation and sarcomere alignment in other *in vitro* cardiac models.^28^^,^^43^ Indeed, our data showed that co-culture EHTs exhibited higher alignment scores than CM-only EHTs from across both cell lines. Well-organized sarcomere networks aligned along the axis of contraction support more efficient force generation, providing a structural basis for the elevated contractility. Previously, our lab demonstrated that atrial fibrillation hiPSC-CMs display disorganized sarcomere compared to their isogenic controls.^44^ Similarly, hypertrophic cardiomyopathy models have also reported sarcomere disarray in disease EHTs compared with controls.^45^^,^^46^ The improved sarcomere alignment observed in co-culture EHTs at baseline may enhance their capacity to detect disease-associated sarcomere disorganization, potentially supporting their utility for cardiac disease modeling.

### Electrophysiological properties of co-culture EHTs

The restitution curves of co-culture EHTs, with steeper slopes, more closely resemble the physiological restitution curves from an adult human heart.^47^ In addition, co-culture EHTs display features of maturation and incorporate CFs, making them a more physiologically accurate platform for drug testing. Twenty drugs with clinically known QT-prolonging effects have been systematically tested in the CiPA initiative using hiPSC-CMs,^48^^,^^49^ which could be improved by incorporating more cell types that are present in the human heart. EHTs have also been shown to reliably detect drug-induced APD changes and respond robustly to targeted ion channel modulation.^5^^,^^50^ Meanwhile, another study has demonstrated that drug responses vary with the maturation state of the cells^51^: mature hiPSC-CMs respond differently to drug-induced APD prolongation compared to more fetal-like cells.

Moreover, cardiotoxic drugs such as doxorubicin have direct effects on CFs. Research has shown that doxorubicin induces fibrosis in CFs^52^ and trigger reactive oxygen species (ROS) production.^53^ It also reduces fibroblast proliferation and migration—processes that are critical for cardiac tissue repair following injury.^53^ Therefore, incorporating CFs into co-culture EHTs is essential for drug screening, as CMs alone cannot fully capture these CF-mediated aspects of cardiotoxicity. Arrhythmogenic features such as electrical alternans were observed in both CM-only and co-culture EHTs, however, the frequency of these events was lower. This suggests that the incorporation of CFs in the co-culture EHTs could be protective, thus acting as a more stable and representative baseline to compare drug toxicities or disease states.

### Co-culture EHTs were more stress resistant than CM-only EHTs

We observed that co-culture EHTs performed better under stress, showing higher contractile force when paced at faster rates. To investigate the underlying mechanism, we conducted hypoxia testing. Strikingly, the hypoxic level in co-culture EHTs stayed relatively stable at increasing pacing rates, whereas CM-only EHTs became increasingly and progressively hypoxic when paced at 150 beats per minute for 1 hour. This suggests that the co-culture tissues are more resistant to stress due to improved oxygen handling and metabolism. In line with these observations, quantitative metabolomics revealed upregulated levels of the hypoxia markers, hypoxanthine and malic acid, in CM-only EHTs.^54^^,^^55^ Given that myocardial contraction is highly oxygen-dependent,^23^^,^^56^ these findings also provide a mechanistic explanation for the higher force generation of co-culture EHTs, particularly at higher pacing rates.

### Co-culture EHTs exhibited improved fatty acid metabolism

The adult heart relies primarily on fatty acids as its main energy source and generates most ATP through oxidative phosphorylation.^23^^,^^56^ Previous studies have shown that 3-dimensional culture of hiPSC-CMs improves mitochondrial structural and promotes expression of mitochondrial proteins similar to those seen in an adult human heart,^57^ which favors oxidative metabolism and fatty acid oxidation over glycolysis. Metabolomics analysis revealed that several metabolites involved in fatty acid transport were upregulated in CM-only EHTs. These intermediates are critical components of the fatty acid oxidation pathway. Accumulation of those intermediates may indicate impaired mitochondrial transport or incomplete β-oxidation, suggesting inefficiency in fatty acid utilization as an energy source.^58^^,^^59^  l-Carnitine and nine acylcarnitines (LCACs), including three short chain and six long chain LCACs, are upregulated in CM-only EHTs compared to co-culture EHTs. Elevated circulating acylcarnitine levels have been reported in heart failure patients,^59^^,^^60^ consistent with incomplete fatty acid oxidation.^61^ Moreover, LCAC accumulation has been linked to hypertrophic cardiomyopathy severity,^60^ and increased arrhythmogenicity.^62^ Pantothenate, a precursor to CoA, was also elevated in CM-only EHTs,^63^ further suggesting dysregulated fatty acid metabolism. Overall, EHTs composed solely of CMs exhibit disease-like characteristics, whereas co-culture EHTs demonstrate improved metabolic function and more closely resemble healthy controls.

In addition, metabolites related to oxidative stress response were upregulated in CM-only EHTs compared to co-culture EHTs. Glutathione**—**an antioxidant that neutralizes ROS,^64^ and its precursors (glycine, glutamate and S-adenosyl-methionine) were elevated, consistent with enhanced glutaminolysis under oxidative stress.^65^ Other antioxidants, including mannitol and taurine, were also increased in CM-only EHTs. Mannitol is known to have osmoprotective properties and capable of scavenging ROS.^66^ Taurine has anti-inflammatory effects and may be protective against coronary heart disease at high levels.^67^ Increased mannitol and taurine levels may reflect an adaptive response to elevated oxidative stress. Together, these findings indicate that CM-only EHTs experienced higher oxidative stress, and in response activated and upregulated compensatory antioxidant pathways, whereas co-culture EHTs appeared to be more stable with less antioxidant stress behavior. Finally, we recognize that our metabolomics analysis was limited by the fact that a large proportion of differentially expressed metabolites remain unidentified. Most metabolites upregulated in co-culture EHTs were unknown, which restricted our ability to draw definitive mechanistic conclusions. Expanding sample size and improved metabolite annotation using our publicly available data set in future studies will increase the power and interpretability of these and other analyses.

### Co-culture EHTs showed reduced variance

Of critical importance was the observation that the APD measurements of co-culture EHTs exhibited smaller variance compared to CM-only EHTs in both cell lines. Co-culture EHTs also showed reduced variance across a series of hypoxia inducing conditions, replicating their protective effect with minimal interexperiment or intraexperimental variability compared to CM-only EHTs. To date, the field of hiPSC-CM have been plagued by inter-lab, inter-line and batch to batch variability which has limited its translational potential.^68–70^ Our co-culture EHTs offer the solution with a more reliable and reproducible translational model with reduced variability, further supporting the potential of co-culture EHTs as a safer and more predictive platform.

## Conclusions

Incorporating hiPSC-CFs into EHTs led to both structural and functional improvements. Compared to CM-only EHTs, our co-culture EHTs generated higher contractile force, displayed more organized sarcomere structure, and demonstrated enhanced metabolic function. Together, they resulted in less variability, reproducible and translational physiology that mimicked the human adult state, and improved resistance to stress conditions. Our co-culture EHT model can be relied on as a more physiological and predictive preclinical platform for cardiac disease modeling and drug screening.

## Supplementary Material

szag041_Supplementary_Data

## Data Availability

The data generated or analyzed during this study are available from the corresponding author on reasonable request.
