# Supplementary material for: Co-culturing hiPSC-cardiomyocytes and cardiac fibroblasts enhances engineered heart tissue structure and function
Source: Stem Cells Transl Med. 2026 Jun 27;15(7):szag041. doi: 10.1093/stcltm/szag041 (PMC13387363; doi:10.1093/stcltm/szag041)
Supplement: szag041_Supplementary_Data [file szag041_supplementary_data.docx]

**Title:**

Co-culturing hiPSC-cardiomyocytes and cardiac fibroblasts enhances engineered heart tissue structure and function

**Running head:** Co-culture EHT as a physiological pre-clinical model

**Authors:** Yinhan Luo^a,b^, BSc, Jeremy Parker^a,c^, PhD, Armando Alcázar Magaña^d^, PhD, Ardin Sacayanan^a,c^, BASc, Kate Huang^a,b,e^, PhD, Ian Fernandes^f,g^, PhD, Gordon M. Keller^f,g^, PhD, Peter Backx^h^, PhD, Leonard J. Foster^i^, PhD, Zachary Laksman^a,b,c,e^, MD, MSc, FRCPC

**Affiliations:**

1. Centre for Heart Lung Innovation, University of British Columbia, Vancouver, Canada
2. Department of Experimental Medicine, University of British Columbia, Vancouver, Canada
3. School of Biomedical Engineering, University of British Columbia, Vancouver, Canada
4. Michael Smith Laboratories, Department of Biochemistry & Molecular Biology, University of British Columbia, Vancouver, Canada
5. Division of Cardiology, Department of Medicine, University of British Columbia, Vancouver, Canada
6. McEwen Stem Cell Institute, University Health Network, Toronto, Canada
7. University of Toronto, Toronto, Canada
8. Department of Biology, York University, Toronto, Canada
9. Life Sciences Institute, University of British Columbia, Vancouver, Canada

**Author contributions:**

Yinhan Luo: Conception and design, collection and/or assembly of data, data analysis and interpretation, manuscript writing

Jeremy Parker: Provision of study material or patients, data analysis and interpretation

Armando Alcázar Magaña: Collection and/or assembly of data, data analysis and interpretation

Ardin Sacayanan: Data analysis and interpretation, manuscript editing

Kate Huang: Manuscript editing

Ian Fernandes: Provided cardiac fibroblasts and differentiation resources

Gordon M. Keller: Provided resources and supervision

Peter Backx: Provided resources and supervision

Leonard J. Foster: Provided resources and supervision

Zachary Laksman: conception and design, manuscript editing, final approval of manuscript

**Funding and support:** This work was supported by the Canadian Institutes of Health Research (CIHR) AWD-029166, Stem Cell Network IMP-C4R2-10 and Genome BC CBIC 197PCO.

**Conflict of interest:** The authors declare no conflicts of interests.

**Address for correspondence:** Dr. Zachary Laksman MD, MSc, FRCPC

zlaksman@mail.ubc.ca

Rm. 220 – 1033 Davie Street, Vancouver, BC V6E 1M7

Phone: 604-806-8256 Fax: 604-806-8723

**Supplementary Information**

**Table of contents**

1. **Methods………………………………………………………………………………………3-8**
2. **Supplemental Table 1. RT-qPCR primer sequences………………………………………..9**
3. **Supplemental Table 2. Levene’s test results of level of hypoxia……………………………10**
4. **Supplemental Table 3. Levene’s test results of APD80 from line 1 EHTs…………………10**
5. **Supplemental Table 4. Differentially expressed metabolites between CM and CMCF EHTs in line 1………………………………………………………………………………….11**
6. **Supplemental Table 5. Differentially expressed metabolites between CM and CMCF EHTs in line 2………………………………………………………………………………….12**
7. **Supplemental Figure 1. hiPSC-CM and hiPSC-CF differentiation efficiency and marker expression………………………………………………………………………………………13**
8. **Supplemental Figure 2. Assessment of the contractility and sarcomere alignment of CM-only and co-culture EHTs……………………………………………………………………..14**
9. **Supplemental Figure 3. Electrophysiological characterization of CM-only versus co-culture EHTs…………………………………………………………………………………..15**
10. **Supplemental Figure 4. Metabolic profiling of CM-only versus co-culture EHTs………..16**

**Methods:**

***hiPSC-CM differentiation***

Previously characterized male and female hiPSC lines from healthy donors were maintained in mTeSR1 medium (STEMCELL Technologies) on hESC-qualified Matrigel (Corning). Cells were passaged with ReLeSR™ (STEMCELL Technologies) at 70%–80% confluency and reseeded at a 1:50 ratio in mTeSR1 supplemented with 10 μM Y-27632 (R&D Systems). CM differentiation was induced by temporal Wnt pathway modulation using a small-molecule protocol.^23–25^ Cells were cultured in RPMI 1640 (MilliporeSigma) with B27 minus insulin (Thermo Fisher Scientific) from day 0 to day 6, with 12–14 μM CHIR99021 (STEMCELL Technologies) added on day 0 and 7.5 μM IWP2 (Selleck Chemicals) added 24 hours later. From day 7, cells were maintained in RPMI 1640 with B27 supplement (Thermo Fisher Scientific). Lactate purification was performed from day 10 to day 15 using glucose-free RPMI 1640 containing 5 mM sodium L-lactate (Sigma-Aldrich). Freshly differentiated hiPSC-CMs were used for EHT generation.

***hiPSC-CF differentiation***

hiPSCs were differentiated to CFs through sequential Wnt modulation and FGF2 (Miltenyi Biotec) treatment.^26^ Cells were cultured in RPMI 1640 on Matrigel coated plates from day 0 to day 6, with 6 μM CHIR99021 added on day 0 and 2.5 μM IWP2 added on day 3. On day 6, cells were split at a 1:6 ratio and seeded on 0.1% gelatin (Sigma-Aldrich)-coated plates in LaSR (RPMI 1640 supplemented with GlutaMAX [Invitrogen] and ascorbic acid [Sigma-Aldrich]). From day 7 to 12, cells were cultured in LaSR and treated with 3μM of CHIR99021 on days 7 and 8. On day 12, cells were split at a ratio of 1:10 and cultured in LaSR containing 20 ng/mL FGF2 from day 12 to 18. hiPSC-CFs were then passaged and expanded in Fibroblast Growth Medium 3 (PromoCell) and cryopreserved.

***Engineered heart tissue generation***

EHTs were generated using the fibrin-based hydrogel method,^27^ and the tools for EHT casting were purchased from DiNABIOS. Agarose molds were cast in a Nunc 24-well plate, followed by placement of polydimethylsiloxane (PDMS) posts. The hydrogel master mix consisted of DMEM/F-12 (Gibco), B27 supplement, GlutaMAX, water for injection, penicillin-streptomycin, approximately 1 mg/mL of Matrigel and 5.4 mg/mL of fibrinogen (Sigma-Aldrich). For four EHTs, 3.2x10^6^ hiPSC-CMs (CM-only EHTs) or 2.4x10^6^ hiPSC-CMs and 0.8x10^6^ hiPSC-CFs (co-culture EHTs) were resuspended in 400 μL hydrogel and 1μL of 10mM Y-27632. 12 μL of thrombin (Sigma-Aldrich) was added immediately before casting and 100 μL (containing approximately 0.8x10^6^ cells) was dispensed into each mold well.

Plates were incubated at 37°C with 5% CO_2_ for 1 hr to allow tissue formation. 500 μL of EHT culture medium was added into each well to detach tissues from the mold. The EHTs were incubated for another 20 min before being transferred to fresh plates. EHTs culture medium consisted of DEME/F-12 supplemented with B27 supplement, 33 μg/mL of aprotinin (Sigma-Aldrich), and 50 U penicillin-streptomycin. From day 6 to day 12, maturation supplements were added, including triiodothyronine (Sigma-Aldrich), dexamethasone (Sigma-Aldrich), oleic acid-BSA conjugate (Sigma-Aldrich) and palmitic acid-BSA conjugate (Cedarlane Labs).^6^ Media were performed every other day, and spontaneous contractions were typically observed within one week of casting.

***Flow cytometry***

hiPSC-CMs were dissociated into single cells by incubation in RPMI 1640 supplemented with 0.5 U/mL Liberase TH (Sigma-Aldrich), 50 U/mL DNaseI (Sigma-Aldrich), and TrypLE (Thermo Fisher Scientific) at 37°C. The cells were fixed with 2% paraformaldehyde (BD BioSciences) at room temperature (RT) and permeabilized with 0.5% saponin and 2% FBS in DPBS-/-. Cells were stained with BV421-conjugated anti-cTnT antibody (BD BioSciences) for 30 min at RT. hiPSCs were dissociated with Versene (Gibco) and stained in parallel as negative controls. Samples were analyzed on a Gallios Flow Cytometer (Beckman Coulter) and data were processed using the Kaluza Analysis Software v2.1 (Beckman Coulter).

***RT-qPCR***

RNA was extracted from cell pellets using the Single Cell RNA Purification kit (Norgen Biotek). and Animal Tissue RNA Purification kit (Norgen Biotek). 500 ng of RNA was reverse transcribed to cDNA using the All-In-One 5X RT MasterMix (Applied Biological Materials). 15 ng of cDNA was combined with 500 nM of primers (Integrated DNA Technologies; Supplemental Table 1) and PowerUp SYBR Green Master Mix (Thermo Fisher Scientific). qPCR was performed on QuantStudio 6 Pro Real-Time PCR system (Thermo Fisher Scientific) and analyzed using the Design and Analysis Software v2.4.3. Relative gene expression was calculated using the ΔCT method with *GAPDH* and *HSA36B4* as the reference genes.

***EHT cross-sectional area measurement***

EHTs were processed on the Leica ASP 6025 automated vacuum tissue processor. They were fixed in 10% neutral buffered formalin for 24 hrs and immersed in 70% and 90% ethanol for 1 hr each for dehydration. EHTs then went through three changes of 100% ethanol (1 hr each), three changes of xylene (45 min each) and three changes of paraffin (1 hr each). They were embedded in paraffin, sliced at 4 µm along the transverse plane, and stained with hematoxylin and eosin. EHTs were imaged at 10X using EVOS 3000 automated cell imager. Cross-sectional area of the EHTs was measured using ImageJ and the average areas were used to normalize contractile force.

***Immunofluorescence***

Cells were fixed with 2% paraformaldehyde for 10 min at 37°C, permeabilized with 0.2% Triton X-100 in DPBS-/- for 15 min at RT and blocked for 1hr in 3% bovine serum albumin (Sigma-Aldrich). hiPSC-CMs were stained with anti-α-actinin (Sigma-Aldrich) and anti-titin-M line (Myomedix), and hiPSC-CFs were stained with anti-vimentin and anti-zonula occludens-1 antibodies. Cells were then incubated in goat anti-mouse AF488 (Abcam) and goat anti-rabbit AF647 (Thermo Fisher Scientific) secondary antibodies and Hoechst 33342 (Cedarlane Labs) for 1 hr at RT. 63X images of stained cells were acquired with a Zeiss LSM 880 Confocal Microscope with Zen Black 2.3 SP1 FP1 version 14.0.9.201.

***Sarcomere alignment visualization and quantification***

EHTs were incubated in 30 mM of 2,3-butanedione monoxime (Sigma-Aldrich) diluted in DPBS-/- at 37°C for 10 min. Then, EHTs were removed from the silicone posts and fixed with 4% paraformaldehyde for 1 hr. They were permeabilized with 1% Triton X-100 for 1 hr and blocked with 3% bovine serum albumin for 3 hrs. EHTs were stained with mouse anti-α-actinin (Sigma-Aldrich) antibody overnight at 4°C and goat anti-mouse secondary antibody (Abcam) and Hoechst 33342 for 3 hrs at RT. EHTs were transferred onto glass-bottom dishes for imaging with the confocal microscope. 63X images were taken along the length of each EHT at seven positions and at three different focal planes. Images with the clearest and brightest sarcomere were analyzed using the scanning gradient Fourier transform (SGFT) method^28^ in MATLAB version R2022b.

***Contractile force measurement***

A 10-second recording of the EHT was acquired using a Nikon Ti2-E microscope equipped with a pco.edge 4.2Q High QE sCMOS camera, as previously described.^23^ The EHTs were electrically stimulated at increasing rates and a video was recorded at each rate using a 4X objective. A custom MATLAB-based analysis software was used to calculate the contractile force generated by the EHTs. The post deflection was detected, and the displacement was converted into force using MATLAB’s peak detection function and known properties of the PDMS posts. Contractile force was then normalized to the cross-sectional areas of the EHTs.

***Optical mapping of voltage and calcium transients***

EHTs were co-stained with FluoVolt (Thermo Fisher Scientific) and Calbryte 630 AM (AAT Bioquest) in DMEM/F12 medium supplemented with 0.02% pluronic F-127 (Thermo Fisher Scientific) and 1.25 μM probenecid (AAT Bioquest). EHTs were imaged in a stage-top incubator at 37°C and 20-second recordings were acquired at 10X, 200 frames per second with 2x2 binning using the same Nikon microscope. To measure the voltage and calcium transients, stepwise field electrical stimulation was applied at an increasing frequency by decreasing the cycle length from 1000 ms to 300 ms with a 100 ms increment. Electrophysiological abnormality was defined as the presence of alternans, early-after depolarizations, or delayed-after depolarizations. To measure the conduction velocity, EHTs were subjected to point stimulation at one end of the tissue at cycle lengths of 1200 ms to 600 ms. Five-second recordings were acquired at 4X, 250 frames per second without binning. Data from recordings with those abnormalities were excluded from the restitution curves. Data was processed and analyzed using a custom-built Python analysis script.

***Hypoxia imaging***

EHTs were incubated with 5 μM of Image-iT Green Hypoxia Reagent (Thermo Fisher Scientific) in EHT culture medium as described by Tu et al.^6^ After 1 hr at 37°C, the dye-containing medium was replaced with fresh medium. EHTs were then paced at 400 ms for 1 hr in a stage-top incubator (37°C), and fluorescence images were acquired using a Nikon Ti2-E microscope (4X objective) at 0, 15, 30, 45 and 60 minutes. Background subtraction was performed in NIS-Elements, and fluorescence intensity was quantified using Fiji ImageJ.

***Metabolomics and differential analysis***

CM-only and co-culture EHTs were detached from the PDMS posts on day 35 and stored at -80°C until analysis. Three CM-only and three co-culture EHTs were collected from line 1. Five CM-only and five co-culture EHTs were collected from line 2. Untargeted metabolomics was performed as previously described.^29^ Briefly, EHTs were homogenized and then analyzed using an Impact II high-resolution mass spectrometer (Bruker Daltonics) coupled to an Elute UHPLC system (Bruker) under both positive and negative electrospray ionization modes. A pooled quality control sample was generated by combining aliquots from all EHTs. Raw data were processed and annotated in Progenesis QI software (v.3.0.7600.27622) with METLIN plugin (v.1.0.7642.33805).^29^ Data normalization, principal component analysis and differential expression analysis were performed in R (v4.5.0) using the *POMA* package, and heatmaps were generated with the *pheatmap* package.

***Data visualization and statistics***

Statistical analyses and plots were generated in R (v4.5.0). Data normality was assessed using Shapiro-Wilk test. For normally distributed data, Student’s *t-test* or Welch’s *t-test* was applied; otherwise, the Wilcoxon rank-sum test as used. Variance was evaluated using Levene’s test. Data are represented as median with interquartile range, and *p* <0.05 was considered statistically significant. Diagrams and visual abstracts were created in PowerPoint using icons from Bioicons.

**Supplemental Table 1. RT-qPCR primer sequences**

| **Target gene** | **Forward primer (5’-3’)** | **Reverse primer (5’-3’)** |
| --- | --- | --- |
| *TNNT2* | TTCACCAAAGATCTGCTCCT | TACTGGTGTGGAGTGGGTG |
| *TBX5* | CTTGTGATGTTTTCAGAGCC | TTCTCTCTAAAAGCAAGCGT |
| *MYL2* | ACAGGGATGGCTTCATTGAC | CCGCTCCCTTAAGTTTCTCC |
| *IRX4* | TTCCGTTCTGAAGCGTGGTC | TGAAGCAGGCAATTATTGGTGT |
| *VIM* | AGGCAAAGCAGGAGTCCACTGA | ATCTGGCGTTCCAGGGACTCAT |
| *FN1* | TCTCATTCAACAAGAAACCACTG | TTCACGTCTGTCACTTCCACA |
| *COL1A2* | TGATGGAAAAGGAGTTGGACTT | CAGGTCCTTGGAAACCTTGA |
| *POSTN* | TGCCCTTCAACAGATTTTGG | GCAGCCTTTCATTCCTTCC |
| *MYL7* | GCCCAACGTGGTTCTTCCAA | CTCCTCCTCTGGGACACTC |
| *TNNTI3* | TTTGACCTTCGAGGCAAGTTT | CCCGGTTTTCCTTCTCGGTG |
| *SCN5A_f* | TCATGGGCGTATGTATCAGAAAA | GCTTCTTCACAGACTGGAT |
| *GAPDH* | GGTGTGAACCATGAGAAGTATGA | GAGTCCTTCCACGATACCAAAG |
| *HSA36B4* | CTCGCTTCCTGGAGGGTGTCCGC | CTCCACAGACAAGGCCAGGACTCG |

**Supplemental Table 2. Levene’s test results of level of hypoxia**

| **Cell line** | **Time** | **Levene statistic** | **P-value** |
| --- | --- | --- | --- |
| Line 1 | 0min | NA | NA |
| Line 1 | 15min | 6.413 | 0.024 |
| Line 1 | 30min | 3.430 | 0.089 |
| Line 1 | 45min | 6.462 | 0.025 |
| Line 1 | 60min | 4.565 | 0.051 |
| Line 2 | 0min | NA | NA |
| Line 2 | 15min | 0.518 | 0.484 |
| Line 2 | 30min | 0.186 | 0.674 |
| Line 2 | 45min | 0.094 | 0.764 |
| Line 2 | 60min | 0.319 | 0.582 |

**Supplemental Table 3. Levene’s test results of APD80 from line 1 EHTs**

| **Cycle length** | **CM variance** | **CMCF variance** | **Levene statistic** | **P-value** |
| --- | --- | --- | --- | --- |
| 1000 | 0.005 | 0.013 | 1.944 | 0.177 |
| 200 | NA | NA | NA | NA |
| 300 | 0.0003 | 0.0008 | 3.006 | 0.117 |
| 400 | 0.008 | 0.004 | 1.287 | 0.272 |
| 500 | 0.016 | 0.004 | 2.541 | 0.129 |
| 600 | 0.019 | 0.004 | 7.895 | 0.010 |
| 700 | 0.024 | 0.007 | 3.967 | 0.058 |
| 800 | 0.030 | 0.005 | 7.693 | 0.011 |
| 900 | 0.043 | 0.002 | 9.823 | 0.005 |
| spontaneous | 0.067 | 0.002 | 1.503 | 0.239 |

**Supplemental Table 4. Differentially expressed metabolites between CM and CMCF EHTs in line 1**

| **Compound** | **Log2 fold change** | **P-value** | **Adjusted P-pvalue** |
| --- | --- | --- | --- |
| Creatine | -1.28 | 0.000320 | 0.0337 |
| O-Phosphoethanolamine | -1.32 | 0.000610 | 0.0424 |
| Phosphorylcholine | -1.33 | 0.000794 | 0.0468 |
| Glutathione reduced | -1.33 | 0.00135 | 0.0539 |
| Propionylcarnitine | -1.30 | 0.00151 | 0.0570 |
| S-Adenosyl-methionine | -0.966 | 0.00373 | 0.0811 |
| Isobutyryl-carnitine | -1.30 | 0.00719 | 0.100 |
| Pantothenate | -1.06 | 0.00744 | 0.102 |
| Glutamate | -0.903 | 0.00811 | 0.106 |
| N-acetyl-DL-serine | -0.834 | 0.00887 | 0.107 |
| Aminoisobutanoate | -0.889 | 0.00956 | 0.107 |
| S-Pyruvoylglutathione | -1.15 | 0.0114 | 0.112 |
| L-Carnitine | -0.976 | 0.0133 | 0.119 |
| Phenylacetylglycine | -1.13 | 0.0138 | 0.121 |
| Glycine | -0.874 | 0.0140 | 0.121 |
| Allothreonine | -0.909 | 0.0152 | 0.124 |
| Beta-Nicotinamide adenine dinucleotide | -0.894 | 0.0174 | 0.129 |
| Taurine | -0.945 | 0.0187 | 0.134 |
| Malic acid | -0.822 | 0.0206 | 0.138 |
| N-Acetylaspartylglutamic acid | -0.920 | 0.0211 | 0.139 |
| S-Lactoylglutathione | -0.962 | 0.0256 | 0.145 |
| CDP-ethanolamine | -0.744 | 0.0297 | 0.152 |
| Glutathione (oxidized) | -0.732 | 0.0392 | 0.168 |
| Asparagine | -0.754 | 0.0395 | 0.168 |
| Hypoxanthine | 0.729 | 0.0462 | 0.181 |
| Glycerol 3-phosphate | -0.773 | 0.0468 | 0.182 |
| N-acetylaspartate | -0.729 | 0.0470 | 0.182 |

**Supplemental Table 5. Differentially expressed metabolites between CM and CMCF EHTs in line 2**

| **Compound** | **Log2 fold change** | **P-value** | **Adjusted P-value** |
| --- | --- | --- | --- |
| Mannitol | -1.84 | 0.000365 | 0.215 |
| Sn-glycero-3-phosphoethanolamine | 1.82 | 0.000934 | 0.215 |
| 6-Methylnicotinamide | -1.78 | 0.000955 | 0.215 |
| 5-Methoxytryptamine | -1.52 | 0.00231 | 0.215 |
| Linoelaidylcarnitine | -1.76 | 0.00456 | 0.235 |
| 3-Hydroxylinoleoylcarnitine | -1.46 | 0.00616 | 0.254 |
| Glycyl-D-proline | -1.42 | 0.00713 | 0.264 |
| Linoleic acid | -1.56 | 0.00915 | 0.292 |
| Tridecanedioic acid | -1.17 | 0.00960 | 0.292 |
| Citicoline (CDP-choline) | -1.42 | 0.00969 | 0.292 |
| 5-Methylcytidine | -1.28 | 0.0112 | 0.311 |
| Glycerophosphocholine | -1.27 | 0.0129 | 0.330 |
| Carnitine (Dl) hydrochloride | -1.27 | 0.0137 | 0.337 |
| 4-Hydroxyphenyllactic acid | -1.23 | 0.0149 | 0.344 |
| Glycerylphosphorylethanolamine | -1.25 | 0.0168 | 0.347 |
| Gluconic acid | -1.29 | 0.0171 | 0.347 |
| Adenosine-3-monophosphate | 1.20 | 0.0231 | 0.376 |
| (10Z)-12-Hydroxyhexadecenoylcarnitine | -1.22 | 0.0317 | 0.410 |
| CDP-Ethanolamine | -1.09 | 0.0326 | 0.414 |
| Adenosine monophosphate | 1.08 | 0.0334 | 0.416 |
| 4-Hydroxyheptanoylcarnitine | -0.909 | 0.0378 | 0.432 |
| Linoleyl carnitine | -1.19 | 0.0389 | 0.439 |
| Lysophosphatidylcholine 18:2 | -0.982 | 0.0398 | 0.441 |
| Stearoylcarnitine | -1.17 | 0.0400 | 0.441 |
| Methylthioadenosine | -1.00 | 0.0464 | 0.473 |
| Octadecenoylcarnitine | -1.16 | 0.0472 | 0.475 |
| O-Succinyl-L-homoserine | -1.04 | 0.0472 | 0.475 |


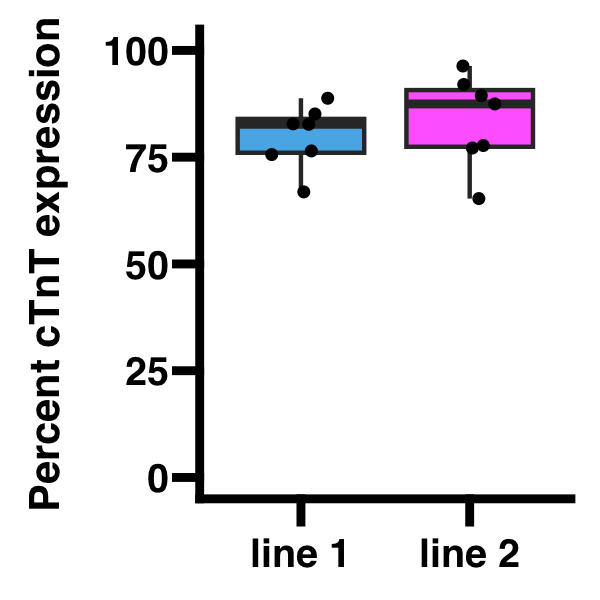


A

B

**
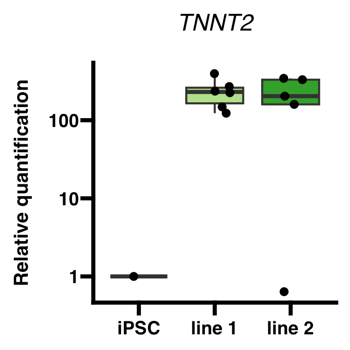
**
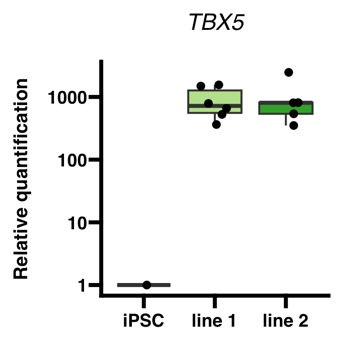

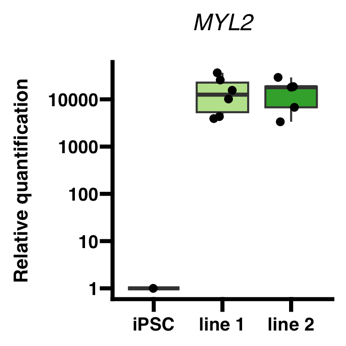
**
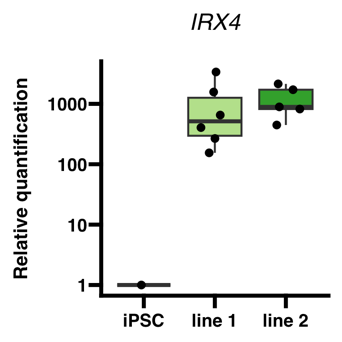
**

C

**
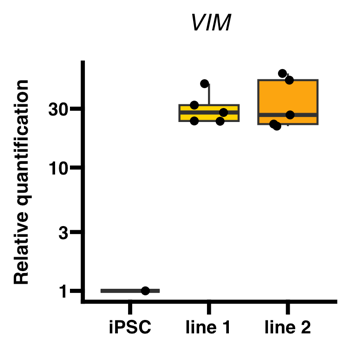

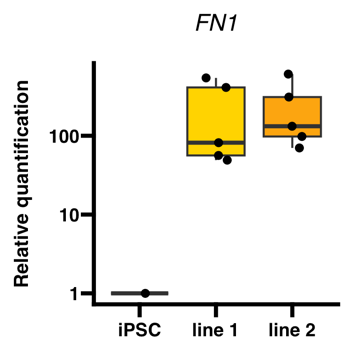

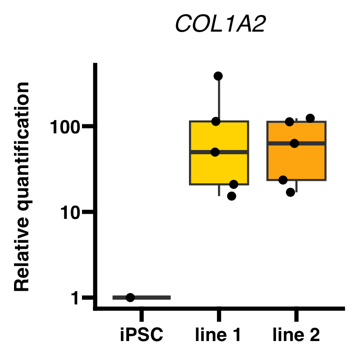

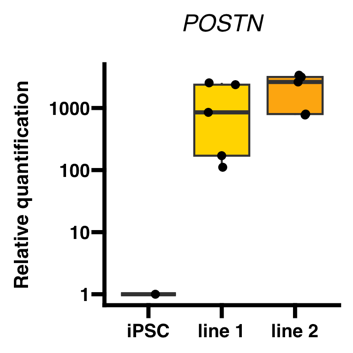
**

**Supplemental Figure 1. hiPSC-CM and hiPSC-CF differentiation efficiency and marker expression.** A) Assessment of hiPSC-CM differentiation efficiency via expression of pan-cardiac marker cardiac troponin T (cTnT) by flow cytometry. B) Assessment of hiPSC-CM gene expression by RT-qPCR: pan-cardiac markers troponin T2, cardiac type (*TNNT2*) and T-box transcription factor 5 (*TBX5*) and ventricular markers myosin light chain 2 (*MYL2*) and Iroquois homeobox 4 (*IRX4*). C) Expression of fibroblast markers in hiPSC-CFs: vimentin (*VIM*), fibronectin 1 (*FN1*), collagen type I alpha 2 chain (*COL1A2*) and periostin (*POSTN*).


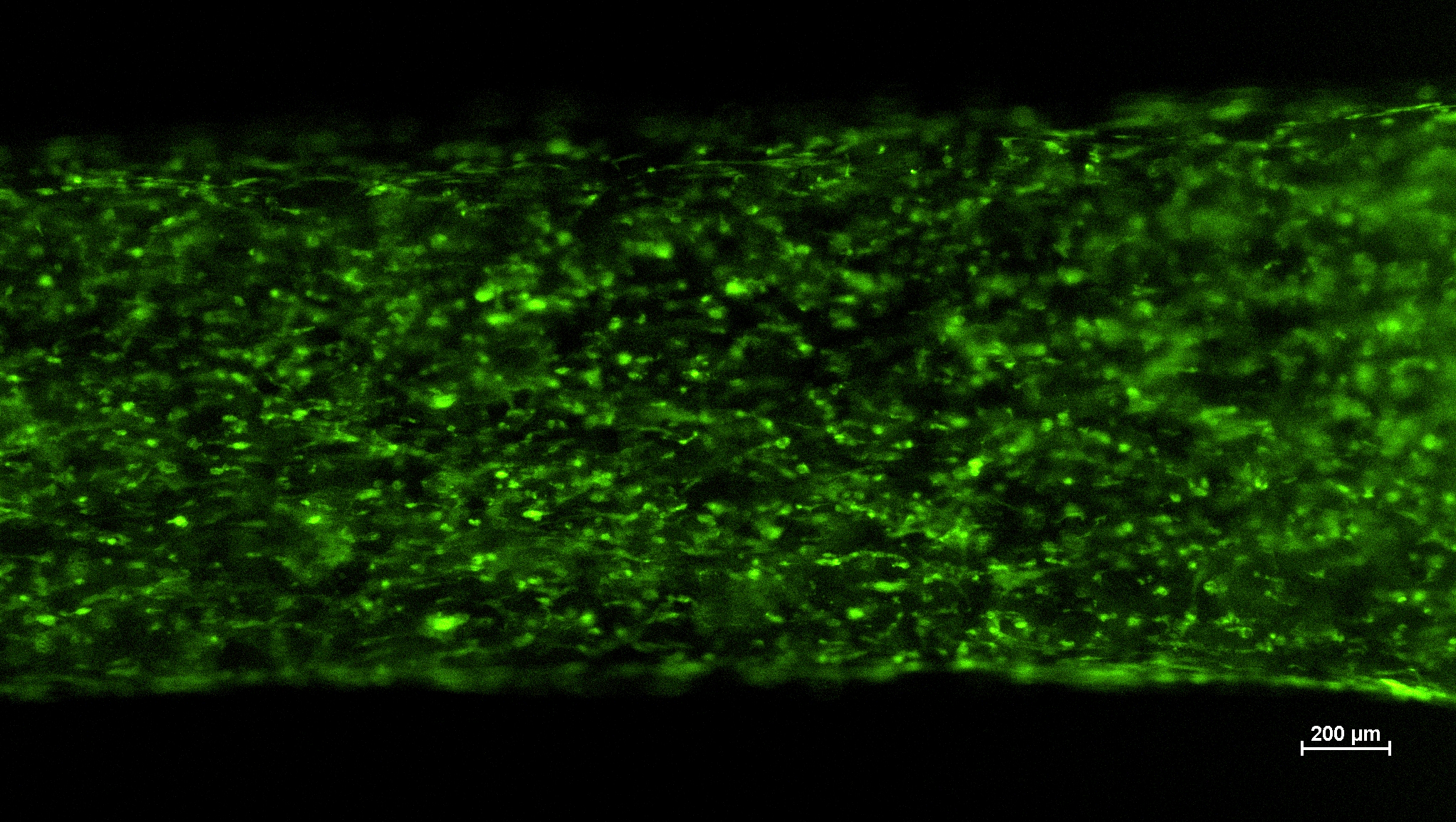

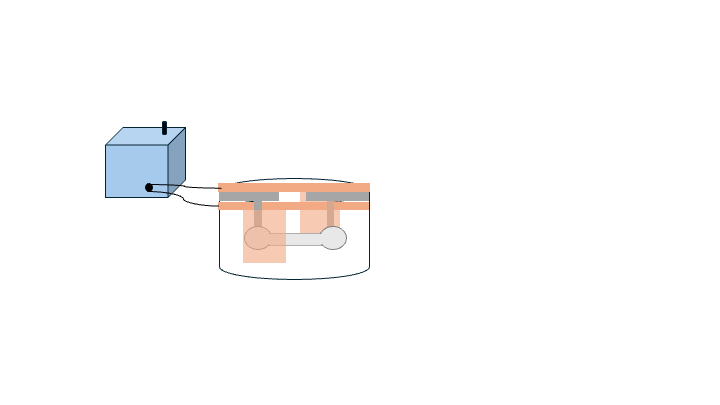


Media

EHT

Carbon plate

Electrode

Stimulator

A

B

D

C

*

***

*

***

**
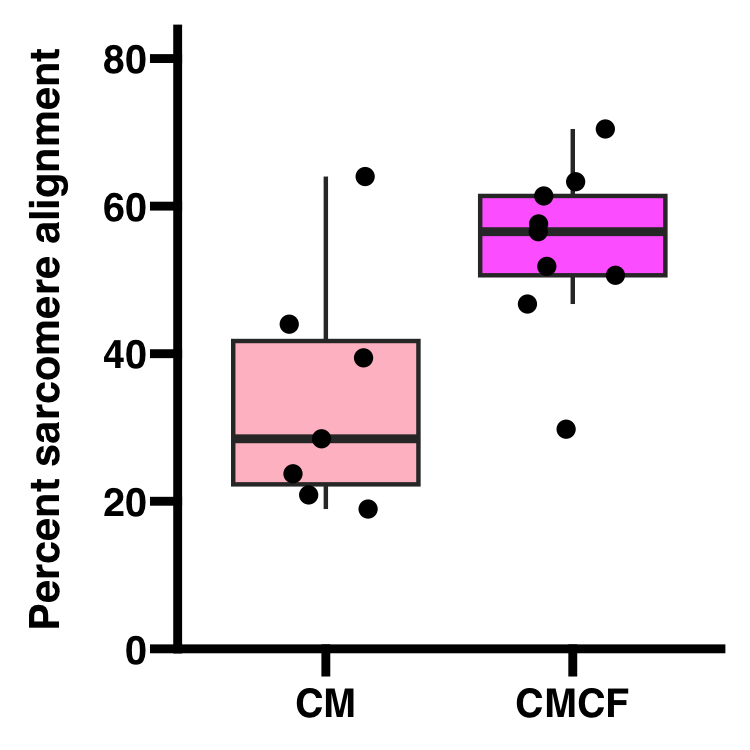

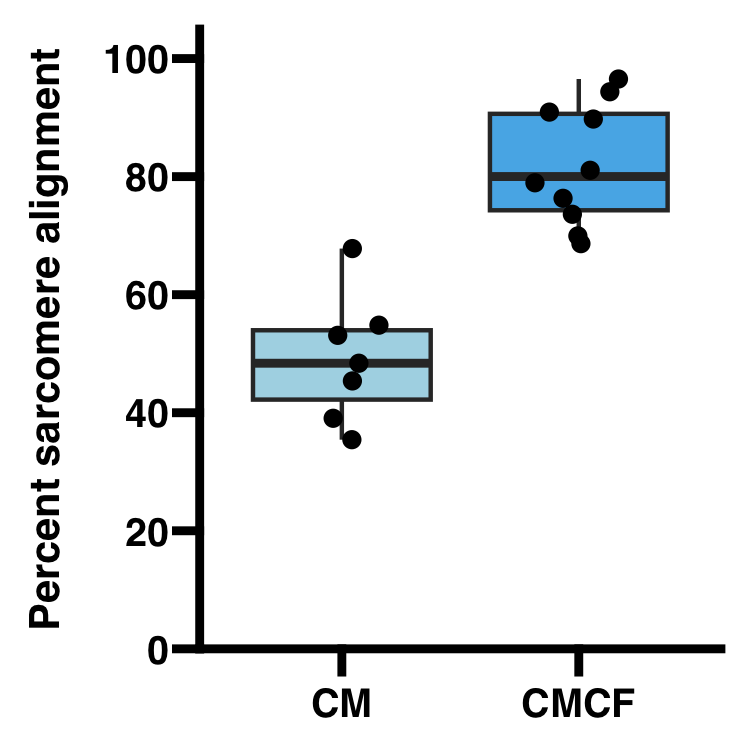

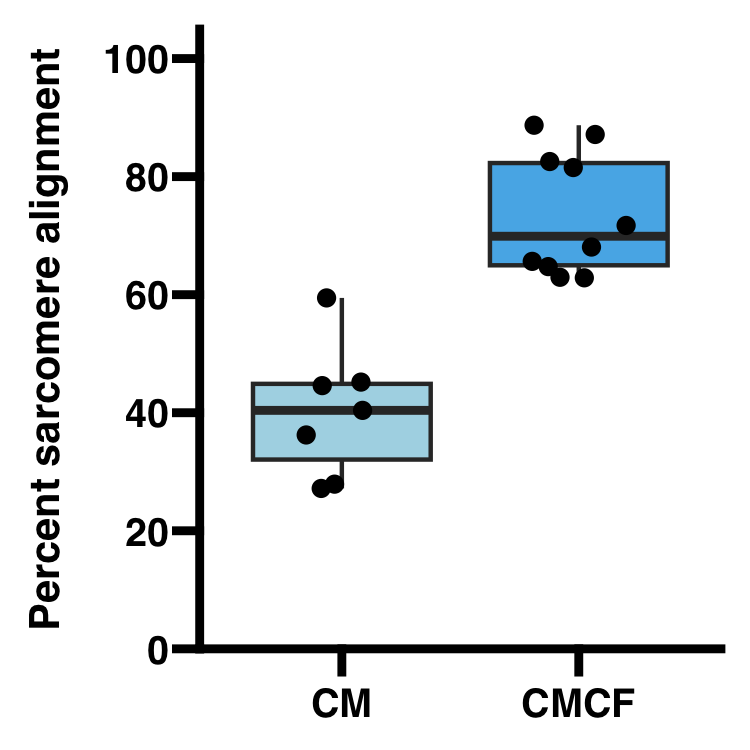

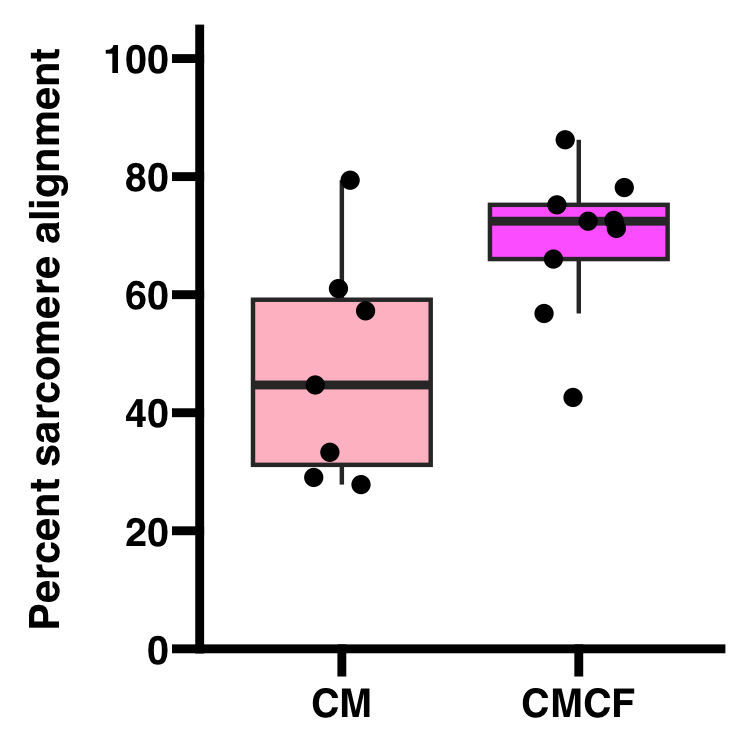
**

**Supplemental Figure 2. Assessment of the contractility and sarcomere alignment of CM-only and co-culture EHTs.** A) Microscopic image of a co-culture EHT with GFP-tagged CFs. B) Electrical stimulation setup: a custom-built stimulator applies a voltage and paces the EHTs at increasing frequencies via carbon electrodes. The EHT-containing plate is placed in a temperature-controlled chamber on an inverted microscope. C) Percent sarcomere alignment within 15 degrees and D) 20 degrees of CM-only versus co-culture EHTs. Blue: line 1, pink: line2. **P<0.05; **P<0.01*. ****P<0.001.*

A

**
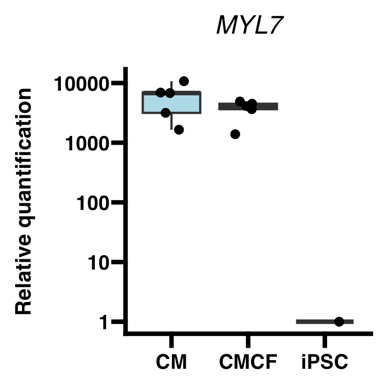

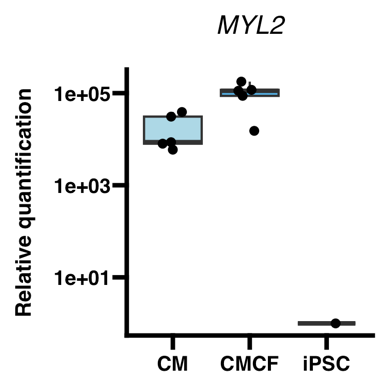

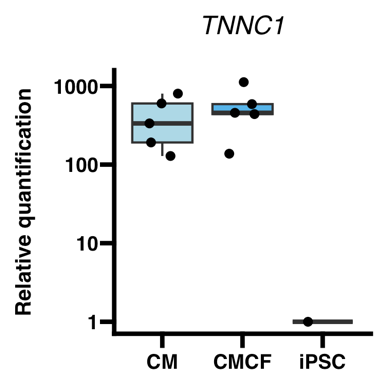

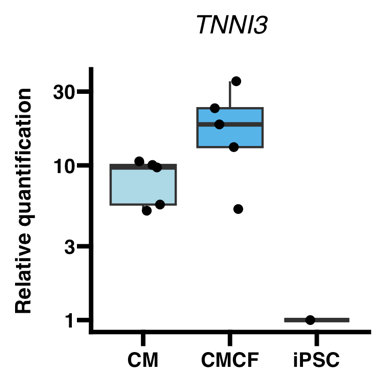
**

ns

ns

ns

ns

B

**
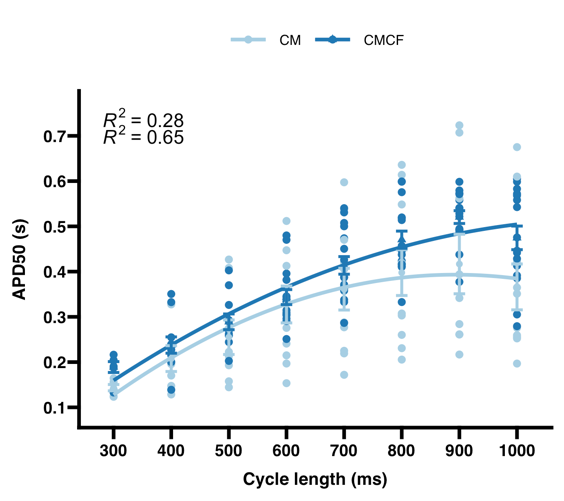

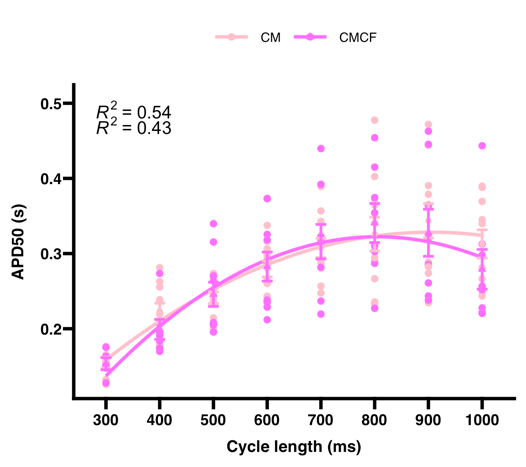
**

**

**

*

C

**
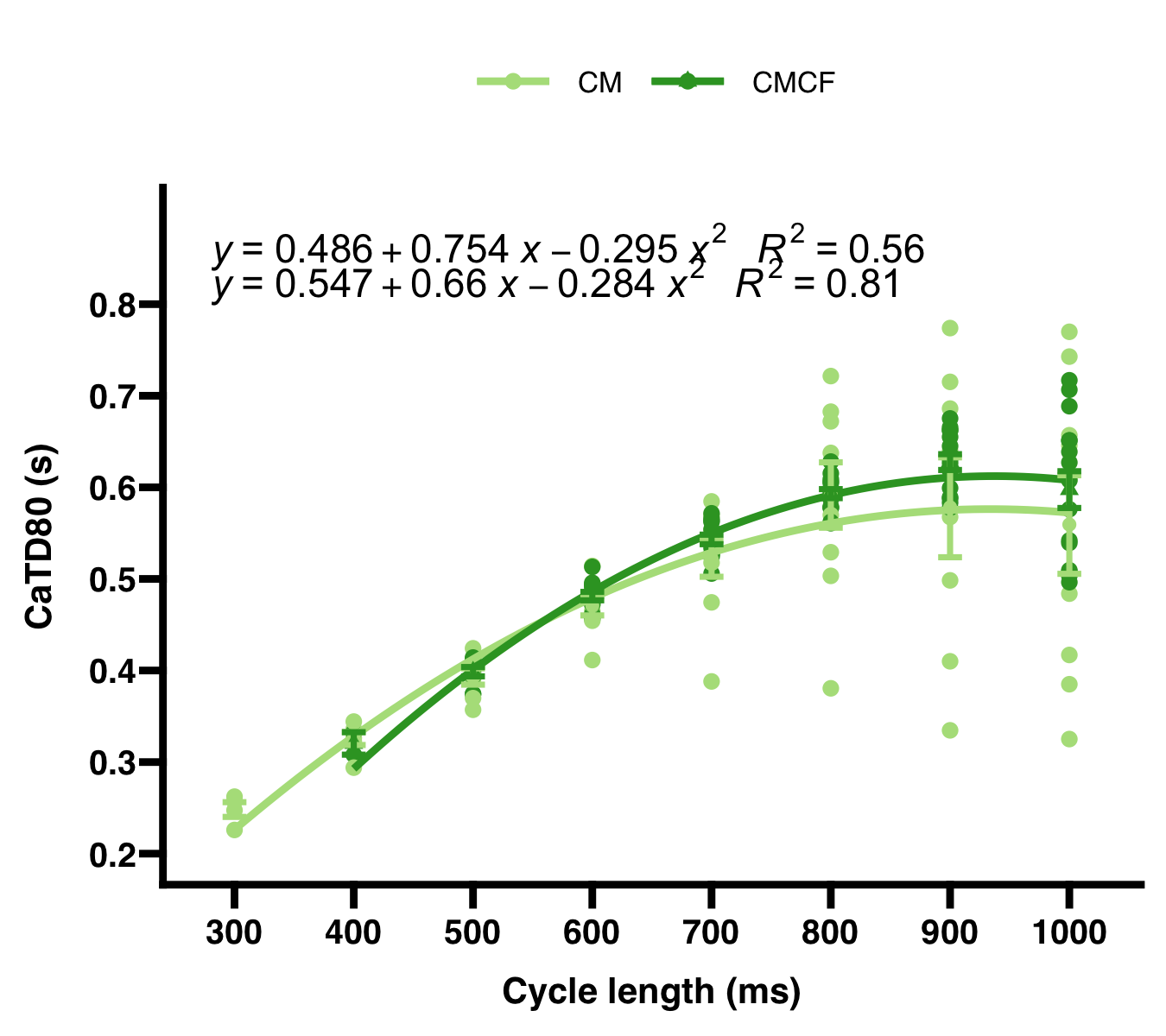

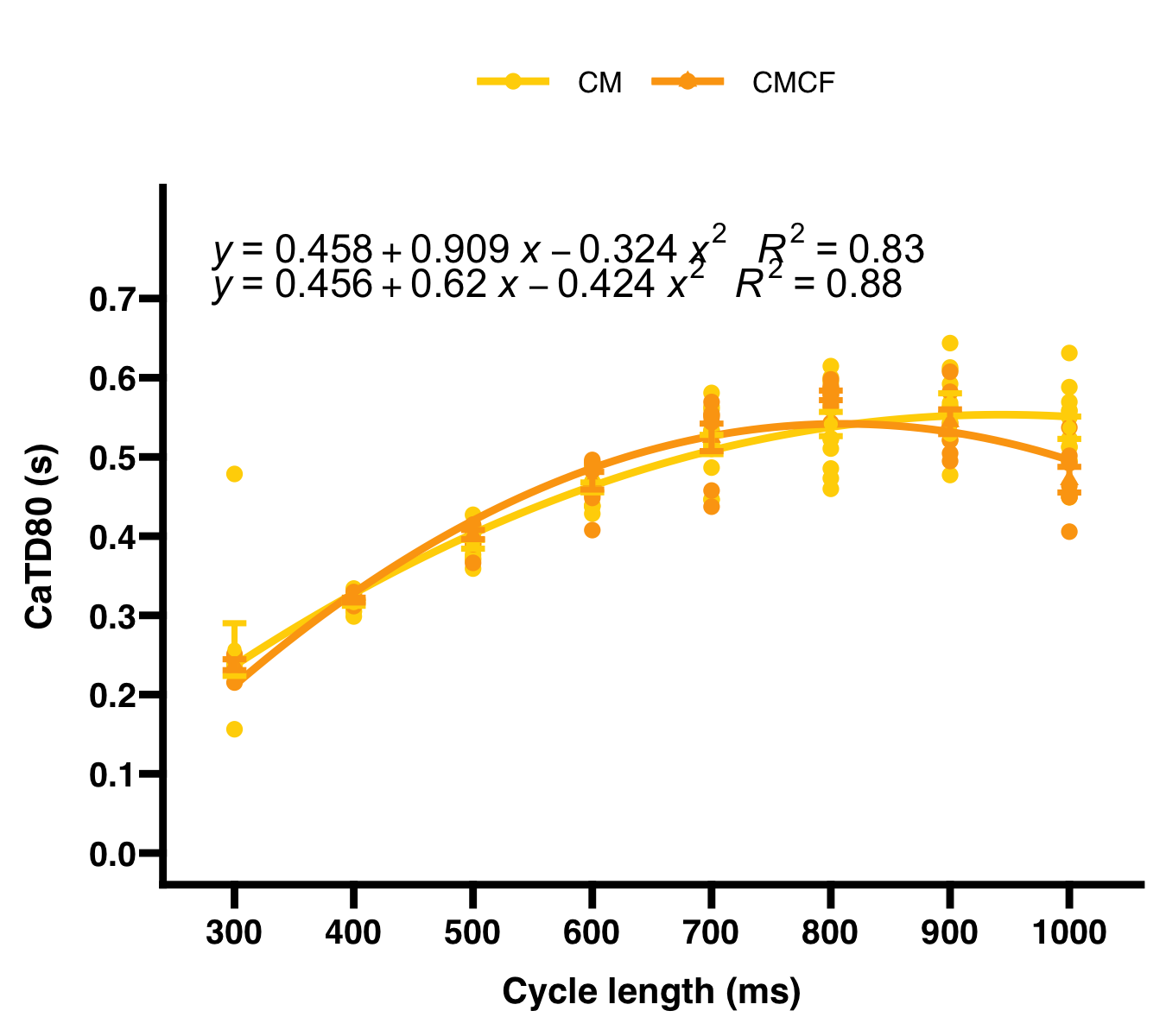
**

**Supplemental Figure 3. Electrophysiological characterization of CM-only versus co-culture EHTs.** A) sarcomere gene and maturation marker expression of CM-only and co-culture EHTs in line 1: myosin light chain 7 (*MYL7*)*, MYL2*, troponin C1 (*TNNC1*) and troponin I3 (*TNNI3*). B) APD50 restitution curves of CM-only versus co-culture EHTs fitted to second degree polynomial equations. Blue: line 1, pink: line 2. C) Calcium transient curves of CM-only versus co-culture EHTs fitted to second degree polynomial equations. Green: line 1, yellow: line 2. **P<0.05. **P<0.01.*

A

B


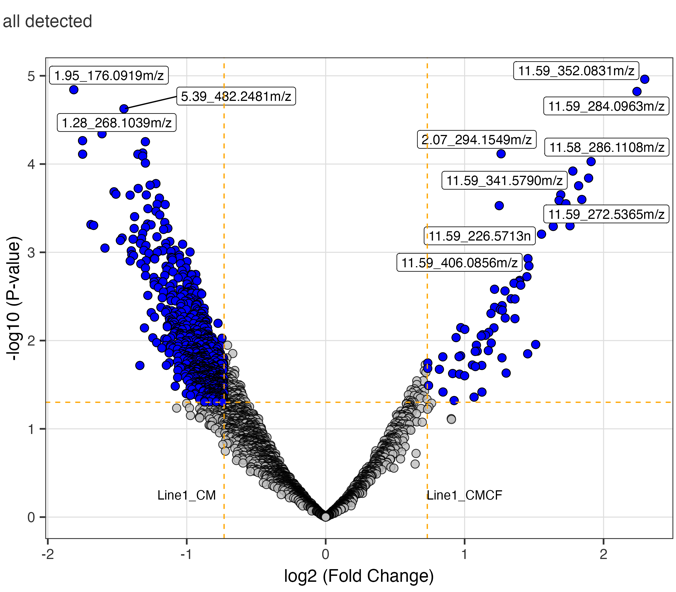

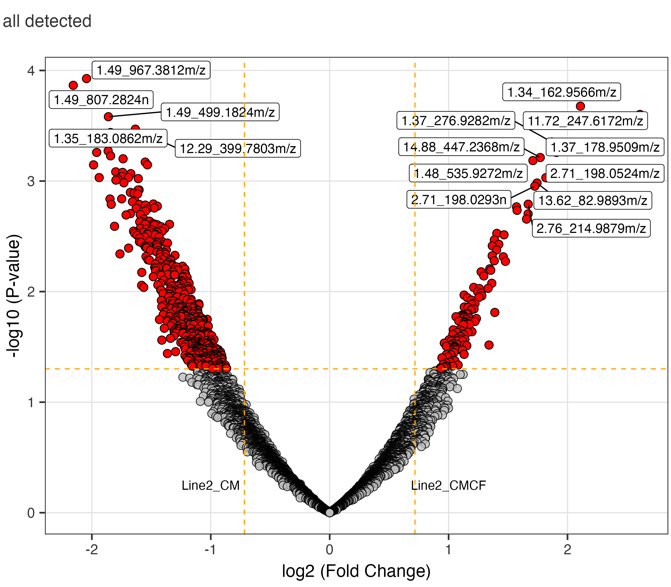


**Supplemental Figure 4. Metabolic profiling of CM-only versus co-culture EHTs.** A) Volcano plots showing all differentially expressed metabolites that were detected between CM-only and co-culture EHTs from line 1 and B) line 2. Blue and red dots represent metabolites with significantly different expression levels.
